# Populational heterogeneity and partial migratory origin of the ventromedial hypothalamic nucleus: genoarchitectonic analysis in the mouse

**DOI:** 10.1007/s00429-022-02601-y

**Published:** 2023-01-04

**Authors:** Lara López-González, Margaret Martínez-de-la-Torre, Luis Puelles

**Affiliations:** University of Murcia, IMIB-Arrixaca Institute of Biomedical Research, El Palmar, 30120 Murcia, Spain

**Keywords:** Ventromedial hypothalamic nucleus, Tuberal hypothalamus, Acroterminal, Tangential migration, Prosomeric model, *Nkx2.2*

## Abstract

The ventromedial hypothalamic nucleus (VMH) is one of the most distinctive hypothalamic tuberal structures, subject of numerous classic and modern functional studies. Commonly, the adult VMH has been divided in several portions, attending to differences in cell aggregation, cell type, connectivity, and function. Consensus VMH partitions in the literature comprise the dorsomedial (VMHdm), and ventrolateral (VMHvl) subnuclei, which are separated by an intermediate or central (VMHc) population (topographic names based on the columnar axis). However, some recent transcriptome analyses have identified a higher number of different cell types in the VMH, suggesting additional subdivisions, as well as the possibility of separate origins. We offer a topologic and genoarchitectonic developmental study of the mouse VMH complex using the prosomeric axis as a reference. We analyzed genes labeling specific VMH subpopulations, with particular focus upon the *Nkx2.2* transcription factor, a marker of the alar-basal boundary territory of the prosencephalon, from where some cells seem to migrate dorsoventrally into VMH. We also identified separate neuroepithelial origins of a *Nr2f1*-positive subpopulation, and a new *Six3*-positive component, as well as subtle differences in origin of *Nr5a1* positive versus *Nkx2.2*-positive cell populations entering dorsoventrally the VMH. Several of these migrating cell types are born in the dorsal tuberal domain and translocate ventralwards to reach the intermediate tuberal domain, where the adult VMH mass is located in the adult. This work provides a more detailed area map on the intrinsic organization of the postmigratory VMH complex, helpful for deeper functional studies of this basal hypothalamic entity.

## Introduction

The classic hypothalamic ventromedial nucleus (VMH) is one of the largest structures in the entire hypothalamus. It was identified within the tuberal area as the “principal nucleus” by Ramón y Cajal (1911). This nucleus contains largely glutamatergic neurons (Ziegler et al. 2002; Puelles et al. 2012) that form several cell masses aggregated in an ovoid block of the tuberal medial hypothalamic stratum surrounded and delimited by a shell of afferent amygdalar inputs (Krieg 1932; Heimer and Nauta 1969). The shell shows non-glutamatergic cell types, in part migrated from the overlying alar plate (Diaz et al. 2015). The VMH is involved in a variety of much studied physiologic functions, which include metabolic regulation (Hetherington and Ranson 1942; Frohman et al. 1974; Elmquist et al. 1999; Dhillon et al. 2006; Kim et al. 2011; Meek et al. 2016), and reproductive (Pfaff and Sakuma 1979a, 1979b; Correa et al. 2015; Hashikawa et al. 2017; Lewis et al. 2022), or aggressive behaviors (Yang et al. 2013; Wang et al. 2015; Kennedy et al. 2020; Hashikawa et al. 2017; Lewis et al. 2022). There is a recent review by Khodai and Luckman (2021). Conventionally, this structure is subdivided in 2–3 parts regarding its aggregation patterns, neuronal morphology, molecular phenotype, and birth dating, normally described topographically as dorsomedial, central, and ventrolateral VMH formations as seen in coronal sections interpreted as cross-sections within the columnar model (Gurdjian 1927; Krieg 1932; Shimada and Nakamura 1973; Altman and Bayer 1978, 1986; McClellan 2006; Kim et al. 2019; van Veen et al. 2020). Introduction of the updated prosomeric model of the hypothalamus by Puelles et al. (2012) provisionally did not change these terms, to avoid confusion, but they are clearly incongruent with the differently oriented prosomeric forebrain axis (e.g., the ‘dorsomedial’ part would be described rather as ‘caudomedial’, and the ‘ventrolateral’ part as ‘rostrolateral’). Though this conventional schema is habitually visualized in a single coronal section plane (i.e., is bidimensional), Van Houten and Brawer (1978), also thinking in columnar terms, contemplated in addition the ‘anteroposterior’ dimension (equivalent to the dorsoventral axis in prosomeric terms). They identified “anterior”, “middle”, and “posterior” differences within the dorsomedial and ventrolateral VMH partitions studied in coronal sections which correspond to the prosomeric dorsoventral topologic differences presented in the present report (see also Table 3).

As regards connections, the VMH nucleus sensu lato is connected reciprocally to the amygdala, septum, preoptic area, paraventricular and anterior (subparaventricular) hypothalamus, contralateral VMH, tuberal, dorsomedial, ventral and dorsal premamillary, medial mamillary and retromamillary nuclei, prethalamic zona incerta, paraventricular and parataenial thalamic nuclei, tegmental ventral area, periaqueductal gray and raphe nuclei (Saper et al. 1976; Canteras et al. 1994; Saper and Lowell 2014; Shimogawa et al. 2015). Many of these areas are differentially innervated by the diverse VMH subdivisions. For instance, forebrain structures regulating the steroid hormonal signaling system, including the medial preoptic, tuberal and ventral premamillary nuclei, receive inputs mainly from the classic ventrolateral VMH subdivision (Canteras et al. 1994). Moreover, the different VMH parts seem associated to different functions. Dorsomedial VMH and central/core VMH were linked to metabolic circuitry (Kim et al. 2011; Meek et al. 2016), whereas ventrolateral VMH controls reproductive and aggressive behavior (Lee et al. 2014; Lin et al. 2011; Hashikawa et al. 2017; Lewis et al. 2022).

**Fig. 1:**
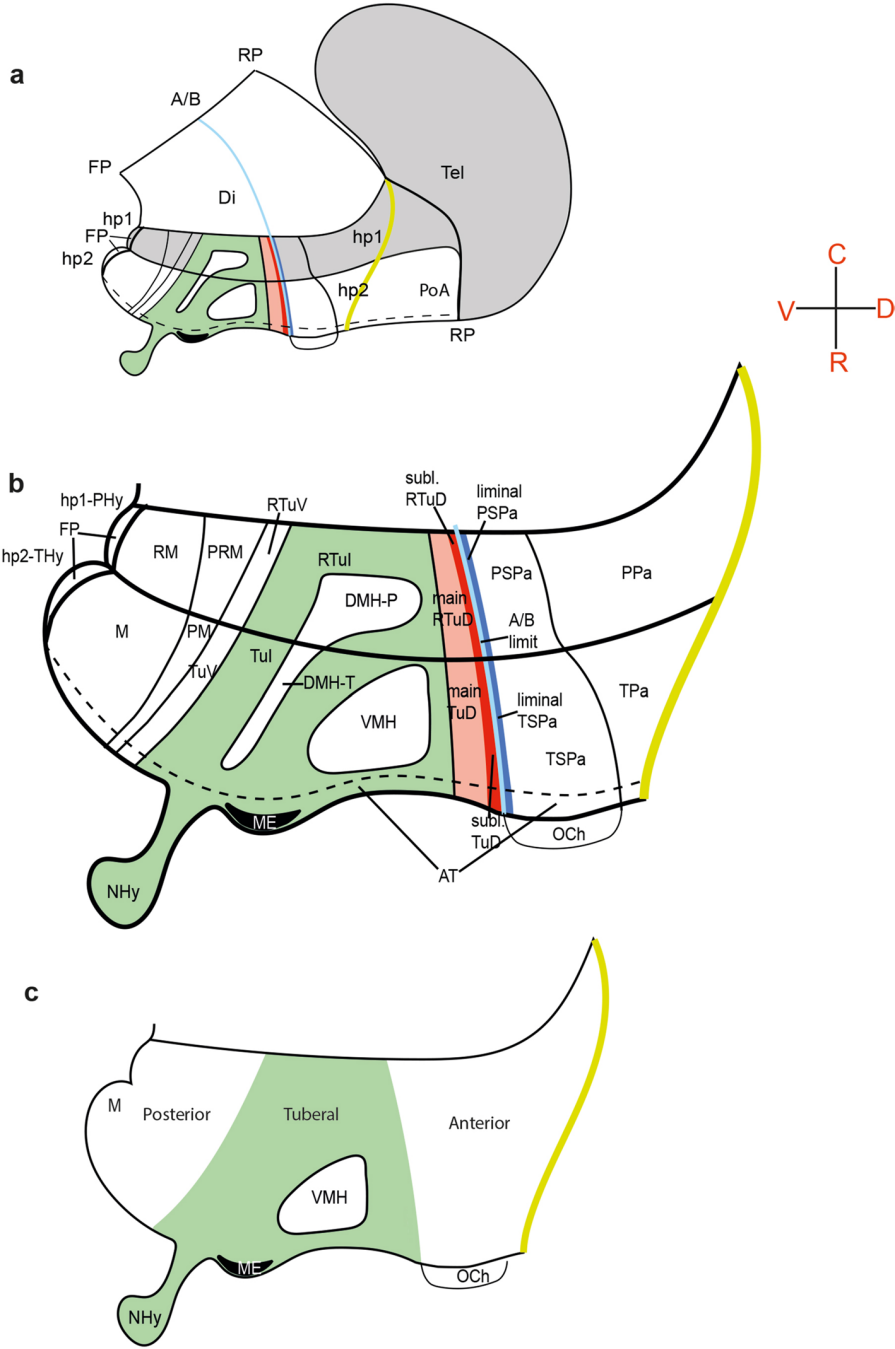
Comparative schemata of the hypothalamus representing: **a**, **b** the prosomeric model and **c** the columnar model. A panoramic view of the position of the hypothalamus in the forebrain is shown in (**a**). **a**, **b** The prosomeric hypothalamus is divided into peduncular and terminal portions (PHy; THy) corresponding to hypothalamo-telencephalic prosomeres hp1 and hp2. In these schemata the yellow line indicates the dorsal longitudinal limit between hypothalamus and telencephalon, whereas the retromamillary (RM) and mamillary (M) areas contact ventrally the hypothalamic floorplate (FP), a primary axial longitudinal landmark induced by the notochord (not shown). The diencephalic and telencephalic roofplate (RP), another primary longitudinal landmark, ends rostrally over the preoptic region (PoA). An intermediate light blue longitudinal line identified as A/B in **a**, **b** represents the postulated alar/basal boundary, parallel to both FP and RP; it is held to result from dorsoventral patterning antagonism between ventralizing floorplate signals and dorsalizing roofplate signals (it is thus a secondary axial landmark shared by all brain parts and supported by many gene expression patterns). The  rostromedian aspect of the entire hypothalamo-preoptic region represents the singular acroterminal domain (AT in **b**), which marks from floor to roof the topologic rostral end of the neural tube. These consistent and causally fundamented axial landmarks justify the spatial orientations provided [R(ostral), C(audal), D(orsal), V(entral)], such that the diencephalon proper lies caudal to the hypothalamus and the telencephalic vesicle is a dorsal outgrowth of the hypothalamus. The eye evaginates out of the alar AT domain (see optic chiasma –OCh– in **b**). **b** This more detailed schema displays hypothalamic structure divided in two transverse neuromeres (hp1/PHy; hp2/THy) extending dorsalward from the floorplate (FP) up to the hypothalamo-telencephalic limit (HTL, marked by a thick yellow line). A  light blue line tagged ‘A/B limit’ marks the longitudinal alar-basal limit which separates the alar and basal hypothalamic progenitor areas. There is a definite AP and DV pattern held to be causally significant. The progenitor domains identified on the basis of differential expression of transcription factors and other molecular markers (Puelles et al. 2012) are thus organized rostrocaudally (relative to the diencephalon, hp1/PHy and hp2/THy) and dorsoventrally (relative to FP, A/B, HTL, and the telencephalic RP). The rostromedian section of the neural tube where right and left brain halves meet is identified as the acroterminal sector (AT). There is a dorsoventral pattern of seven longitudinal progenitor areas (2 alar and 5 basal), each one with its peduncular and terminal part (see list of Abbreviations for the respective areal names). We add some secondary subdivisions such as the liminal PSPa/TSPa alar band and the subliminal RTu/Tu basal band, these being concepts used in the text. The ventromedial nucleus (VMH) is placed in its adult position with its neighbor, the dorsomedial nucleus complex (VMH; DMH-P; DMH-T; within the green RTuI/TuI domain). **c** According to both classical and recent studies the columnar model does not separate PHy from THy and defines its axial or longitudinal dimension as roughly parallel to the prosomeric AT domain, somehow implying an extension of the brainstem and midbrain axis across the diencephalon and the hypothalamus into a telencephalic end (not stipulated precisely). In general, the entire columnar hypothalamus is conceived recently as a floor and basal region of the diencephalon, subdivided by transverse planes into preoptic, anterior, intermediate (tuberal) and posterior (mamillary) regions (Swanson 2012); note the prosomeric AT domain is  conceived rather as prechordal floor. This model classifies the whole prosomeric alar hypothalamus as an Anterior hypothalamic region (containing both paraventricular and anterior hypothalamic nuclei). The prosomeric basal hypothalamus corresponds to the tuberal and mamillary parts of the columnar hypothalamus. The spatial orientations in this model are rotated 90 degrees relative to the prosomeric ones (compare **b** with **c**) *R* rostral, *C* caudal, *D* dorsal, *V* ventral, *Di* diencephalon, *Tel* telencephalon, *hp1* hypothalamic prosomere 1, *hp2* hypothalamic prosomere 2, *FP* floor plate, *PoA* preoptic area, *PHy* peduncular hypothalamus, *THy* terminal hypothalamus, *RM* retromamillary area, *M* mamillary area, *PRM* periretromamillary area, *PM* perimamillary area, *RTuV* retrotuberal ventral area, *TuV* tuberal ventral area, *RTuI* retrotuberal intermediate area, *TuI* tuberal intermediate area, *NHy* neurohypophysis, *ME* medial eminence, *DMH-P* peduncular part of the dorsomedial hypothalamic nucleus, *DMH-T* terminal part of the dorsomedial hypothalamic nucleus, *VMH* ventromedial hypothalamic nucleus, *RTuD* (main or subl.), retrotuberal dorsal area (main or subliminal); TuD (main or subl.), tuberal dorsal area (main or subliminal); *AT* acroterminal area, A/B limit, alar/basal limit; *PSPa* peduncular subparaventricular area, *TSPa* terminal subparaventricular area, *OCh* optic chiasma, *PPa* peduncular paraventricular area, *TPa* terminal paraventricular area

These antecedents, taken jointly with some molecular developmental aspects reviewed in Puelles et al. (2012), suggest that the VMH subdivisions may relate to subtly different origins of these neuronal subpopulations, with some relevant progenitor domains possibly lying dorsally or rostrally to the place where the VMH nucleus develops (descriptors according to the columnar paradigm; see Fig. 1). The global cytoarchitectonic boundary that delimits the VMH complex may be due largely to the VMH shell plexus formed around it. Most studies addressing the development of the VMH have used the columnar model of the brain as a morphologic reference (Herrick 1910, 1933; Kuhlenbeck 1973; Alvarez-Bolado et al. 1995), and essentially employed coronal sections through the nucleus, which was assumed to develop in its adult position.

Altman and Bayer (1986), in their autoradiographic neurogenetic study of the rat hypothalamus, identified the ventrolateral VMH as the earliest generated region, followed by the dorsomedial VMH (called ‘dorsalis’ by these authors), whereas their ‘VMH pars basalis’ (possibly referring to the local periventricular stratum) is the last to become postmitotic, in a sequence ranging between E13 and E17. However, these authors did not identify precisely the neuroepithelial origin of the VMH subdivisions and apparently assumed a local radial origin for all of them. A previous autoradiographic study of Shimada and Nakamura (1973) reported the birthdate interval for VMH neurons in the mouse between E10-E14, but only vaguely ascribed their origin to the underlying neuroepithelium. Ulterior studies described some radial migratory cell movements in cultured coronal VMH slices (Dellovade et al. 2000, 2001; McClellan et al. 2006, 2008). Only Puelles et al. (2012; pp 285–287) seem to have considered the possibility of tangential migrations being involved in the development of this nuclear complex.

Some molecular markers specific of different VMH regions have been reported (Kurrasch et al. 2007). Recently cell line reporter studies (for *Shh*, *Gli*, *Neurog2*, *Ascl1*) followed partially some VMH subpopulations and identified the positions they occupy in the adult VMH (Corman et al. 2018; Aslanpour et al. 2020a, b). Nevertheless, confusion persists, unfortunately, since the widely used coronal section plane is usually understood within the columnar model as demonstrating *transversal* relationships. In contrast, conventional coronal sections are roughly *horizontal* in the prosomeric model due to the latter’s different axial references (e.g., the alar-basal boundary and the floor plate) ending in the acroterminal region of the hypothalamus (Puelles et al. 2012).

In this work we have examined the origin of molecularly defined cells populating different VMH subdivisions based on the detailed topologic developmental map of differentially specified progenitor domains available within the updated prosomeric model (Puelles et al. 2012; Puelles and Rubenstein 2015; Puelles 2018, 2019). Our material includes sagittal, horizontal, and transversal sections oriented according to the ‘natural’ prosomeric axis (defined as parallel to the alar-basal boundary, the floorplate, and the precociously underlying notochord; Fig. 1). We complemented these data with some tracing experiments in organotypic cultures of embryonic hypothalamus to demonstrate the reality of dorsoventral migratory displacements predicted by Puelles et al. (2012). We indeed found that different VMH subpopulations are born in different basal tuberal histogenetic progenitor areas, either coinciding with the final VMH locus (only radial migration involved) or placed *dorsal* and/or *rostral* to the VMH ventricular zone, thus implying significant short-range tangential migrations. Some components seem to originate in the overlying alar plate. In particular, we provide additional evidence for the tangential dorsoventral migration of the *Nkx2.2*-expressing VMH cell populations, complementing the material previously commented by Puelles et al. (2012).

## Material and methods

### Allen atlas brain database

We selected several significative gene expression images from the Allen Developing Mouse Brain Atlas (https://developingmouse.brain-map.org/). Whereas we analyzed all mouse material available in this database for each gene selected, we chose images from E13.5, E15.5, E18.5, P1 and P4 to build the figures. Some of then combine Allen Atlas material join with our lab material. We interpreted the Atlas coronal section planes as horizontal (i.e., parallel to the hypothalamic alar-basal boundary shown in Fig. 1b (A/B limit).

### Animals

We studied mouse specimens from several stages of development processed for in situ hybridization or immunohistochemistry techniques: E12.5 (*n* = 2), E13.5 (*n *= 1), E14.5 (*n *= 8), E16.5 (*n* = 5), E18.5 (*n* = 5), adults (*n* = 2). We used E12.5 mice for migration assays (*n* = 6, see section 2.5). The morning in which a vaginal plug was detected was considered as E0.5 in all embryos. Pregnant females were sacrificed by cervical dislocation after inhalation of isofluorane, and then the embryos were extracted. Embryonic brains were dissected out after anesthesia on ice followed by decapitation. For adult animals, after standard sodium pentobarbital anesthesia, the mice were perfused with 4% paraformaldehyde. The brains were dissected out and fixed overnight in 4% paraformaldehyde in pH 7.4 phosphate-buffered saline (PBS) at 4 °C. After washing, they were embedded in 4% agarose in PBS for sectioning. Vibratome sections were obtained 100 μm-thick for ISH or ISH followed by DAB-immunohistochemistry, or 50 mμ-thick for immunoreactions.

### Immunohistochemistry

We performed free floating immunostaining of vibratome sections. For immunofluorescence reaction, sections were washed in PBS-T (PBS-0.3% Triton X-100), blocked (3% BSA in PBS-T, 1–3 h), and incubated in the primary antibody solution (diluted in 3% BSA in PBS-T, 48 h, 4 ℃). Following incubation and several PBT washes, the sections were incubated 2 h with the respective fluorochrome-labeled secondary antibodies, either Alexa 488 donkey anti-rabbit or donkey Alexa 594 anti-mouse (ThermoFisher; 1:200, 2 h). For DAB-immunohistochemistry, vibratome sections were washed in PBS, and then treated with 0.1% hydrogen peroxide in PBS for 30 min, in the dark and at room temperature, to inactivate endogenous peroxidase activity. After standard PBS-T washes, and the blocking step (3% BSA in PBS-T, 1–3 h), the floating sections were incubated with the primary antibody for 48 h at 4 ℃. After PBS-T washes we applied a biotinylated goat anti-rabbit or anti-mouse secondary antibody (1:200, 2 h at room temperature; Vector Laboratories, Burlingame, CA, United States), followed by a streptavidin/horseradish peroxidase (HRP) complex (1:200, 2 h; Vectastain-ABC kit; Vector Laboratories, Burlingame, CA, United States). Histochemical detection of the peroxidase activity was carried out using 0.03% diaminobenzidine (DAB) and 0.005% H_2_O_2._ Primary antibodies were used as follows: mouse anti-Nkx2.2 (1.50; DSHB, Ref. 745A5-s), rabbit anti-Couptf1/Nr2f1 (1.200; Abcam, Ref. ab96846), rabbit anti-Nkx2.1 (1:200; Sigma Aldrich, Ref. SAB3500757), rabbit anti-Isl1 (1:200; Abcam, Ref. ab20670), rabbit anti- Otp (1: 200; F. Vaccarino), rabbit anti TH (1:200; Bio-Techne R&D Systems, Ref. NB300-109).

### In situ hybridization

We used the restriction enzymes and polymerases suitable for specific riboprobe synthesis in the presence of digoxigenin- 11-UTP. The hybridization protocol used was according to Shimamura et al. (1994). Mouse cDNA probes used for in situ hybridization were *Nxk2.2* and *Nr5a1* (J.R. Rubenstein), *Otp* (A. Simeone), *Six3* (P. Bovolenta), and *Satb2* (our own lab, NCBI accession number NM_001358580).

### Organotypic cultures

Brains of embryos dissected from skin and other appendages at E12.5 were collected in artificial cerebrospinal fluid (ACSF) solution at pH 7.4 containing: 4 mM KCl, 1.5 CaCl2, 0.75 mM MgCl2, 129 mM NaCl, and 10 mM D-glucose. We dissected the tissue with dissection tweezers discarding meninges and telencephalic vesicles, and opened the neural tube along the midline. We placed separately the two brain halves upon membrane culture inserts (Millicell Millipore, 0.4 mm, PICM0RG50) within small Petri dishes, with the ventricular surface up, contacting the air, and the pial surface touching across the membrane a substrate of MEM-supplemented medium (1% Penicilin/Streptomycine, 0.065% glucose, 0.5% glutamine, and 1% inactivated fetal bovine serum). Explants were acclimatized for 1 h (37 ℃, 5% CO2), and subsequently marked through the ventricular surface with a CMFDA-coated tungsten particle borne on a sharpened tungsten needle (Alifragis et al. 2002; López González et al. 2021), testing diverse labeling loci along the estimated alar-basal boundary of the hypothalamus (i.e., varying the dorsoventral position relative to this limit, and changing also the anteroposterior position along the THy, including its rostromedian acroterminal domain). After two days in culture conditions (37 ℃, 5% CO2), the explants were fixed with cold paraformaldehyde 4% in PBS for 10 min. To check the position of the CMFDA particle, as well as the labeled cells in the mantle layer, all explants were processed for immunofluorescence with the mouse anti-Nkx2.2 antibody (1.50; DSHB, Ref. 745A5-s).

### Image analysis

We scanned the ISH and ISH/IHC images at high resolution with the Aperio ImageScope software (Leica Biosystems). Immunofluorescent (IF) images were obtained from the sectioned brains and the whole-mounted explants using a confocal SP8 Leica microscope. Individual optic sections were 3 µm apart, and image stacks of various *Z* sizes were generated according to the structures of interest. All figures from the Allen Developing Mouse Brain Atlas (https://developingmouse.brain-map.org/) were 180º rotated (nose at the right side) to have the same orientation than in our own lab images. Figures were constructed using ImageJ, Adobe Photoshop and Adobe Illustrator software.

## Results

### Background introduction to the prosomeric hypothalamus

The neurodevelopmental field is performing gradually a paradigm change from the old columnar model (which has proven to have difficulties assimilating and explaining gene expression patterns) and the modern prosomeric model. Given their fundamental discrepancy on the length axis of the forebrain, there is a terminological problem associated (Puelles et al. 2012; Puelles 2019), which momentarily forces translation of both unconciliable terminologies (see our Table 3) under the assumption that causal explanations will only emerge from the prosomeric notions. The prosomeric hypothalamus consists of two prosomeres, hp2 and hp1, which represent its structure transversal to the axis of the forebrain (Fig. 1; the prosomeric axis is co-defined by the notochord, the floorplate, the alar-basal boundary and the roofplate as mutually parallel longitudinal reference landmarks lying at different dorsoventral levels; Puelles 2018; Amat et al. 2022; the optic tract may be taken as another such reference; Puelles 2022). These two units are both hypothalamo-telencephalic in spatial range and contain respectively the terminal hypothalamus which extends into the non-evaginated preoptic telencephalic subpallium (THy in hp2; Fig. 1a, b) and the peduncular hypothalamus that expands into the rest of the telencephalon (PHy in hp1; Fig. 1a, b; the telencephalon is strictly a hypothalamic bilateral dorsal evagination, being thus epihypothalamic and the Hypo-Thalamus is a wrong term, because this brain region lies *rostral* rather than ventral to the thalamus and diencephalon proper). THy also includes in its acroterminal alar part the eye vesicle and eye stalk region. PHy is continuous dorsally through the interventricular foramen with the whole evaginated telencephalic vesicle (Puelles et al. 2012; Puelles and Rubenstein 2015). The right and left hypothalamic sides meet rostrally along the acroterminal area (AT) of THy, which is a singularly differentiated median transverse territory displaying dorsoventral alar and basal structural specializations (terminal lamina, optic chiasma, anterobasal area, arcuate/median eminence area, infundibulum and neurohypophysis, tuberomamillary, and mamillary subregions; Puelles et al. 2012; Puelles and Rubenstein 2015). The hypothalamic dorsoventral zonal pattern (alar and basal subdomains) is continuous caudalwards with the equivalent diencephalic pattern (Puelles et al. 2012; Puelles and Rubenstein 2015; Díaz et al. 2015; Ferran et al. 2015; López-González et al. 2021), with shared tagmatic properties down to the isthmo-mesencephalic boundary (Puelles 2018). The evaginated telencephalon as well as the essentially acroterminal eye are thus held to be alar derivatives of the hypothalamus, evaginated within hp1 and hp2, respectively. Hypothalamic *Shh* expression induced by the underlying notochord initially extends to its floor and basal plates (limited by the alar-basal boundary) but is later lost at the acroterminal infundibular part of the intermediate tuberal basal plate area (TuI), a secondary effect due to adenohypophysial *Tbx3* signals (Trowe et al. 2013). Regionalization is demonstrated further through gene expression patterns characteristic of the mentioned structural divisions (e.g., *Ntn1*, *Lmx1b*, *Lmx1a*, *Foxa1* are markers present in the hypothalamic floor plate; *Six3*, *Fgf10*, *Fgf8, Dlk1* appear in the acroterminal area; *Nkx2.2* is a linear longitudinal marker, expressed in a band along the forebrain alar-basal limit and overlapping slightly both alar and basal domains; the alar overlap domain is known as the ‘liminal band’, meaning the rim of the alar plate, whereas the basal overlap domain is known as the ‘subliminal band’; Puelles et al. 2012; Fig. 1a, b). Detailed genoarchitectonic dorsoventral pattern in the hypothalamus allows to distinguish four alar and five basal domains that extend longitudinally across both THy (hp2) and PHy (hp1) (see Fig. 1a, b, its legend, and the abbreviations list). The paraventricular (Pa) and subparaventricular (SPa) areas are distinct superposed alar plate domains, where Pa has three dorsoventral subdivisions (not relevant here). There is a major tuberal/retrotuberal basal longitudinal complex (Tu/RTu) across hp2 and hp1 and under the alar-basal boundary; it lies dorsal to underlying parallel perimamillary/periretromamillary (PM/PRM) and mamillary/retromamillary (M/RM) longitudinal areal complexes. The large Tu/RTu subdivides dorsoventrally into 3 subdomains, called dorsal, intermediate, and ventral (e.g., TuD, TuI, TuV within hp2; similar for the RTu subdivisions within hp1; Puelles et al. 2012). In the present report we are concerned essentially with the TuD and TuI hp2 basal longitudinal zones, which relate to the VMH nucleus. It should be noted that TuD is itself divided into the dorsal *Nkx2.2*-positive subliminal band (the area of basal overlap of the *Nkx2.2* band) and the underlying *Nkx2.2 *negative ‘main part’ of TuD (Puelles et al. 2012; their Fig. 8.14B).

### Borders of the VMH

**Fig. 2:**
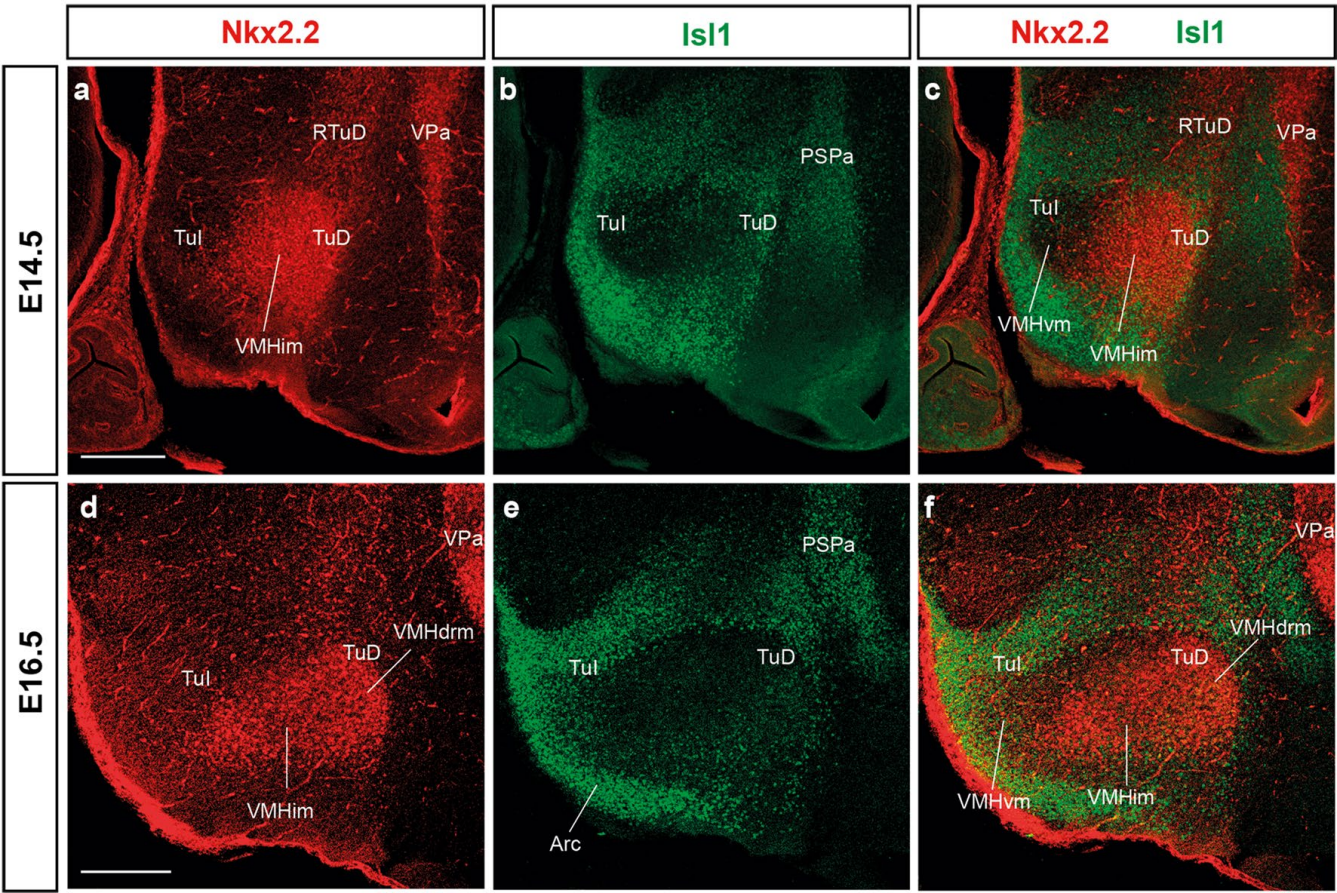
The tuberal/retrotuberal *Isl1* marker delimits negatively the whole VMH primordium early during development. This panel shows sagittal sections of the basal hypothalamus at E14.5 **a**–**c** and E16.5 **d**, **f** labeled with double immunofluorescence for Nkx2.2, and Isl1; the **c**, **f** panels compare the separated red channel **a**, **d** and green channel **b**, **e** images in adjacent sections. The basal areas surrounding (delimiting) the VMH primordium, including the TuI and TuD areas, are marked by intense Isl1 labeling (green), with few Isl1 cells dispersed within the primordium. In contrast, Nkx2.2 labeling (red), while restricted to the VMH primordium, does not fill completely all its volume; note particularly a ventral (VMHvm) triangular sector of the VMH which is also delimited by Isl1 cells, but devoid of Nkx2.2 cells. Scale bars represent 200 µm. *TuI* tuberal intermediate, *RTuD* retrotuberal dorsal, *TuD* tuberal dorsal, *VMHim* medial-intermediate VMH subnucleus; *VPa* ventral paraventricular nucleus, *PSPa* peduncular subparaventricular area, *VMHvm* ventral-medial VMH subnucleus, *Arc* arcuate nucleus, *VMHdrm* dorsal-rostromedial VMH subnucleus

The VMH nucleus occupies a large dorsal area within the TuI region of the terminal hypothalamus, caudal to the acroterminal arcuate nucleus domain (Arc) and dorsal to the terminal part of the dorsomedial nucleus (DM-T); the overlying TuD region contains the wings of the anterobasal nucleus, whose rostromedian fused part is acroterminal (ABasM; ABasW; Fig. 1b; Puelles et al. 2012). The sharp boundary of the VMH proper can be assessed (apart from other possibilities) by its lack of *Isl1* signal, in contrast with the *Isl1*-positive rest of the tuberal region (Fig. 2). Importantly, some VMH neuronal subpopulations seem to have non-TuI origins. They share early molecular markers with cell populations of the overlying TuD region (either its subliminal or main parts), or, alternatively, of neighboring TuD/TuI acroterminal areas, or even of the overlying alar TSPa domain, where some VMH cells apparently originate, as we will illustrate below. Puelles et al. (2012) previously suggested a dorsoventral migration of cells expressing the *Nkx2.2* marker into the VMH primordium.

**Fig. 3:**
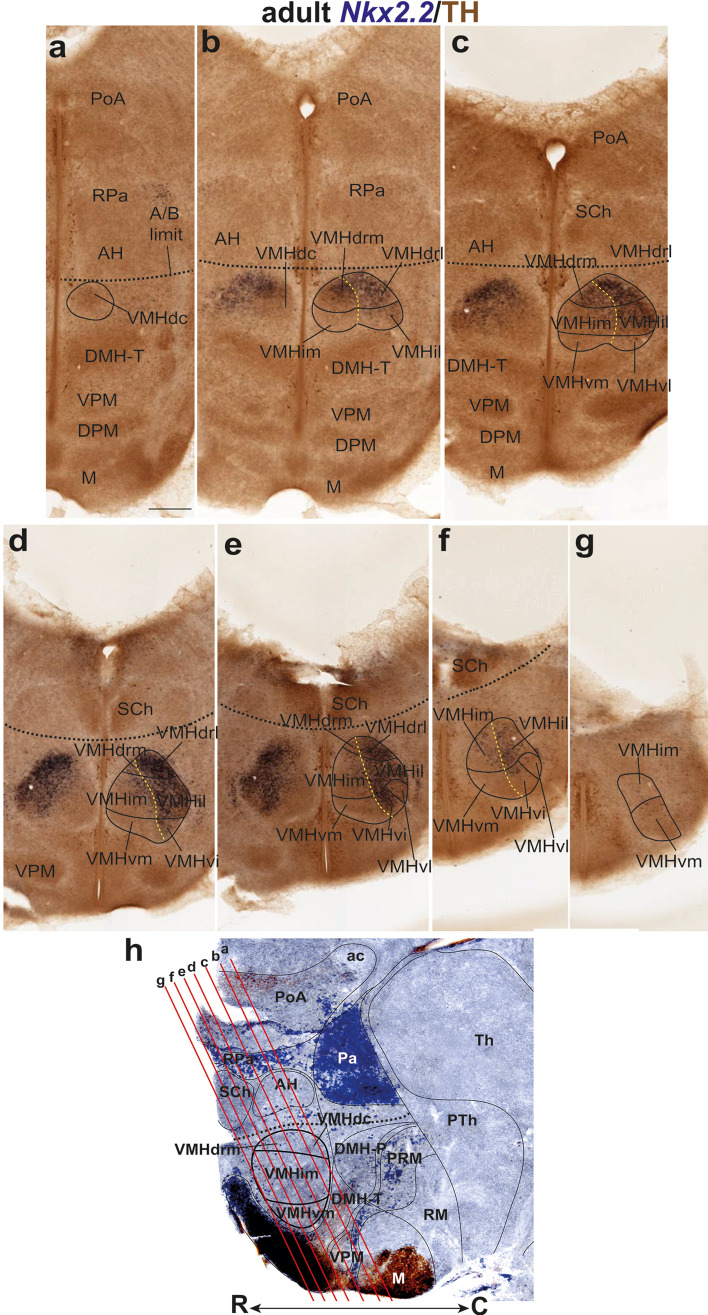
Adult distribution of *Nkx2.2*-positive cells in the VMH. **a**–**g** Caudorostral series of transverse sections orthogonal to the prosomeric forebrain axis taken through the adult terminal hypothalamus to examine systematically the VMH nucleus, and double-reacted for *Nkx2.2* ISH (blue) and TH IHC (brown). The *Nkx2.2*-positive cells are distributed differentially over diverse identified VMH subdivisions. **a** *Nkx2.2* signal is absent at the caudalmost dorsal VMH level, where we distinguish the VMHdc. (**b**–**f**) Progressing rostralward in the series, *Nkx2.2* signal is strongly expressed laterally at VMHdrl and VMHil, and extends into VMHvi; there is also weaker expression at the VMHdrm and VMHim. In contrast, VMHvm, and VMHvl are largely devoid of *Nkx2.2* signal. **g** The rostral-most level of the VMH is also largely *Nkx2.2* negative and can probably be interpreted as VMHim and VMHvm. **h** Sagittal section of the adult medial hypothalamus marked with Nkx2.1 IHC in brown and *Otp* ISH in blue (from Puelles et al. 2012). Red lines represent the section levels in **a**–**g**; see also caudal/C and rostral/R orientations. Scale bars represent 200 µm. *PoA* preoptic area, *RPa* retroparaventricular area, *AH* anterior hypothalamic nucleus; A/B, alar/basal limit, *VMHdc* dorsocaudal subnucleus of the ventromedial hypothalamic nucleus, *DMH-T* terminal part of the dorsomedial hypothalamic nucleus, *VPM* ventral premamillary nucleus, *DPM* dorsal premamillary nucleus, *M* mamillary area, *VMHdrm* dorsal-rostromedial subnucleus of the ventromedial hypothalamic nucleus, *VMHdrl* dorsal-rostrolateral subnucleus of the ventromedial hypothalamic nucleus, *VMHim* medial-intermediate subnucleus of the ventromedial hypothalamic nucleus, *VMHil* lateral-intermediate VMH subnucleu, *SCh* suprachiasmatic nucleus, *VMHvm* ventral-medial VMH subnucleus, *VMHvl* ventral-lateral VMH subnucleus, *VMHvi* ventral-intermediate VMH subnucleus, *ac* anterior comisure, *DMH-P* peduncular part of the dorsomedial hypothalamic nucleus, *PRM* periretromamillary area, *Th* thalamus, *PTh* prethalamus

Our material corroborates the already previously recognized inner heterogeneity of VMH. Dorsomedial, central, and ventrolateral VMH parts are conventionally described in the literature, but we prefer to characterize them as *dorsal*, *intermediate*, and *ventral* regions, respectively (consistently with the prosomeric axial dimension; see Fig. 1). We found that a slightly more detailed subdivision was needed to describe fully the observable molecular diversity. We subdivided the dorsal part of VMH into a *dorsocaudal* element (VMHdc; this is the old ‘dorsomedial’ portion; Fig. 3a, h; see Table 3) and two novel components called by us *medial* and *lateral dorsorostral* subnuclei (VMHdrm, VMHdrl; Fig. 3b–e, h). We also subdivided the intermediate VMH part (classic ‘central’ part) into *medial* and *lateral-intermediate* subnuclei (VMHim, VMHil; Fig. 3b–h). Finally, the large ventral VMH region (old ‘ventrolateral part’) was subdivided into three parts, the *medial, intermediate,* and *lateral ventral subnuclei* (VMHvm, VMHvi, VMHvl; Fig. 3c–h; note the equivalences with the older terminology obviously are not exact; see Table 3).

### Distribution of gene expression during development

We searched genes expressed in mouse TuD and TuI at E13.5 and E18.5 to characterize the expected differential molecular profile of the VMH, using the AGEA tool of the Allen Developing Mouse Brain Atlas database (https://developingmouse.brain-map.org). Tables 1, 2 classify 22 relevant genes according to their characteristic expression domains at E13.5 and E18.5, respectively. Representative markers are shown in Fig. 5, with the respective tridimensional signal distribution presented in medio-laterally ordered sagittal section planes taken from the Allen Developing Mouse Brain Atlas. Although there clearly are areas of overlap between the diverse markers, a dorsoventral, rostrocaudal and mediolateral pattern can be distinguished within the prosomeric model that underpins the detailed topographic subdivision we propose (VMHdc/VMHdrm/VMHdrl; VMHim/VMHil; VMHvm/VMHvi/VMHvl).

**Table 1:** Expression of 22 gene markers at the terminal neurogenic regions of the hypothalamus near the VMH primordium at E13.5

	SPa (Alar)	Subl.TuD	Main TuD	TuI	TuD-AT	TuI-AT
Vax1	+					
Sema3a			+			
Nkx2.2	+	+	+			
Fbxw7			+		+	
Tcf7l2			+		+	
Nr2f1				+		
Lmo4				+		
Satb2				+		
Nr5a1			+	+	+	
Adcyap1			+	+	+	
Robo2			+	+	+	
Calb1			+	+		
Slc17a6			+	+		
Cnr1			+	+	+	
Enc1			+	+	+	
Sox14			+	+	+	
Bcl11a			+	+	+	
Dner			+	+	+	
Chl1			+	+		
Mapt		+	+	+	+	
Nkx2.1			+	+	+	+
Six3					+	+

*SPa* subparaventricular area, *Subl.TuD* subliminal tuberal dorsal area, *Main TuD* main tuberal dorsal area, *TuI* tuberal intermediate area, *TuD-AT* acroterminal section of the tuberal dorsal area, *TuI-AT* acroterminal section of the tuberal intermediate area

**Table 2:** Expression of 22 gene markers within the different VM subnuclei at E18.5

	VMHdc	VMHdrm	VMHdrl	VMHim	VMHil	VMHvm	VMHvl	VMHvi
Vax1		+	+					
Sema3a	+		+					
Nkx2.2		+	+	+	+			+
Fbxw7		+		+				
Tcf7l2		+						
Nr2f1	+					+	+	+
Lmo4				+		+	+	
Satb2	+					+	+	+
Nr5a1	+	+		+				
Adcyap1		+	+	+	+			
Robo2	+	+		+				
Calb1		+		+	+			
Slc17a6	+	+		+	+			
Cnr1	+	+		+			+	
Enc1				+		+	+	
Sox14			+	+		+		+
Bcl11a		+	+			+	+	
Dner		+		+	+	+	+	
Chl1				+	+	+	+	
Mapt	+	+			+	+	+	
Nkx2.1	+	+	+	+		+	+	
Six3		+		+				+

*VMHdc* dorsocaudal VMH subnucleus, *VMHdrm* dorsal-rostromedial VMH subnucleus, *VMHdrl* dorsal-rostrolateral VMH subnucleus, *VMHim* medial-intermediate VMH subnucleus, *VMHil* lateral-intermediate VMH subnucleus, *VMHvm* ventral-medial VMH subnucleus, *VMHvi* ventral-intermediate VMH subnucleus, *VMHvl* ventral-lateral VMH subnucleus

It is possible to cluster the markers studied at E13.5 into five positionally distinct subgroups (alar, only TuD, only TuI, TuD + TuI, only acroterminal; Table 1). These results can be compared with the respective VMH expression pattern at E18.5 (Table 2).

**Fig. 4:**
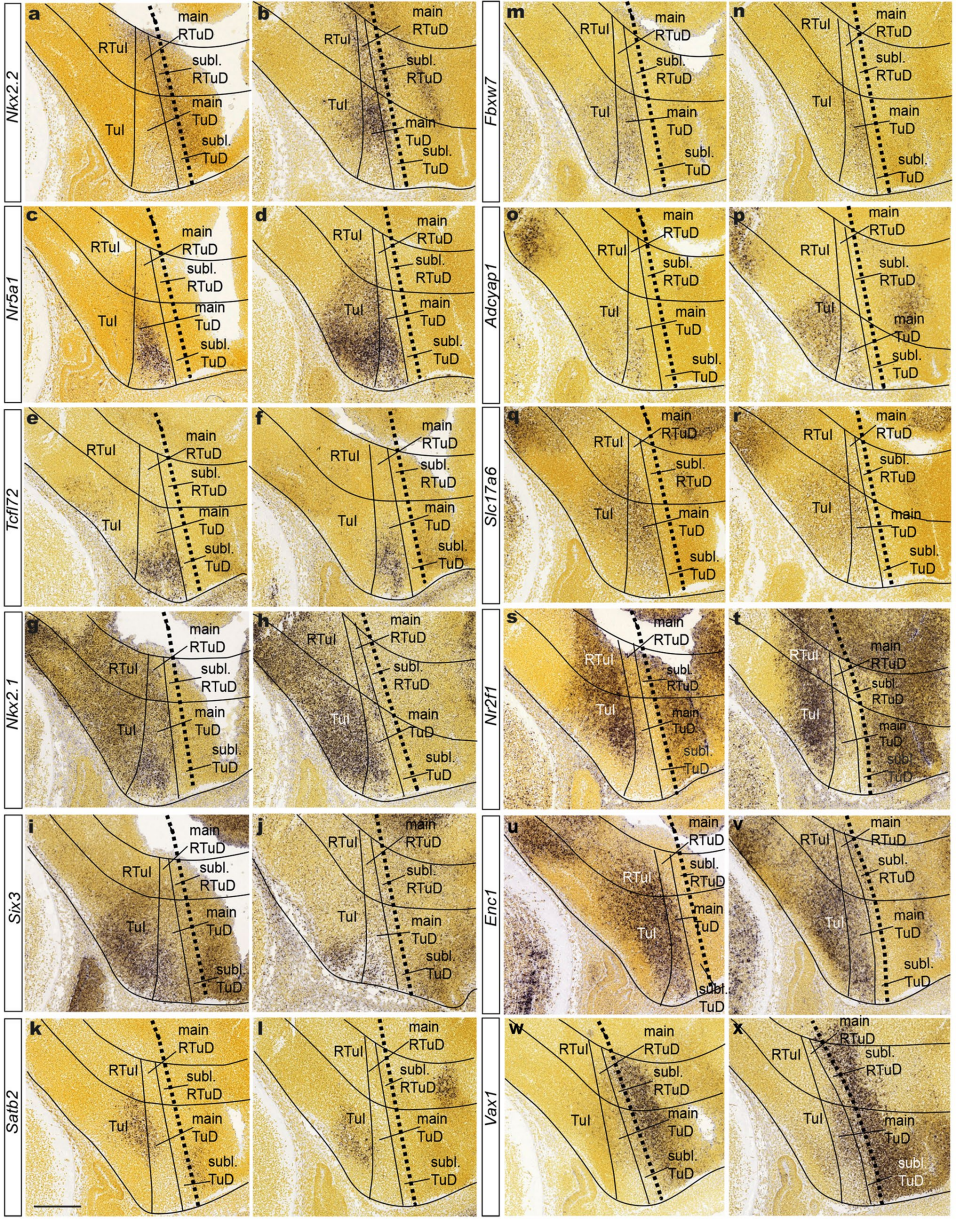
Differential dorsoventral expression of VMH markers at E13.5 in two neighboring sections for each marker (identified at left). This panel illustrates various sagittal ISH images of markers listed in our Tables 1, 2 taken from E13.5 mice material at the Allen Developing Mouse Brain Atlas. The dashed black line identifies the alar-basal boundary (identifying D to the right and V to the left; R to the bottom). TuI, main TuD and subliminal TuD are delimited by solid lines. Other hypothalamic domains are not identified, being outside the scope of this analysis. The observed patterns vary in the degree in which TuI versus TuD, or both, show expression at this stage. Scale bar in **k** represents 200 µm. *RTuI* retrotuberal intermediate area, *TuI* tuberal intermediate area, *main or subl*. *RTuD* main or subliminal retrotuberal dorsal area, *main or subl. TuD* main or subliminal tuberal dorsal area

**Fig. 5:**
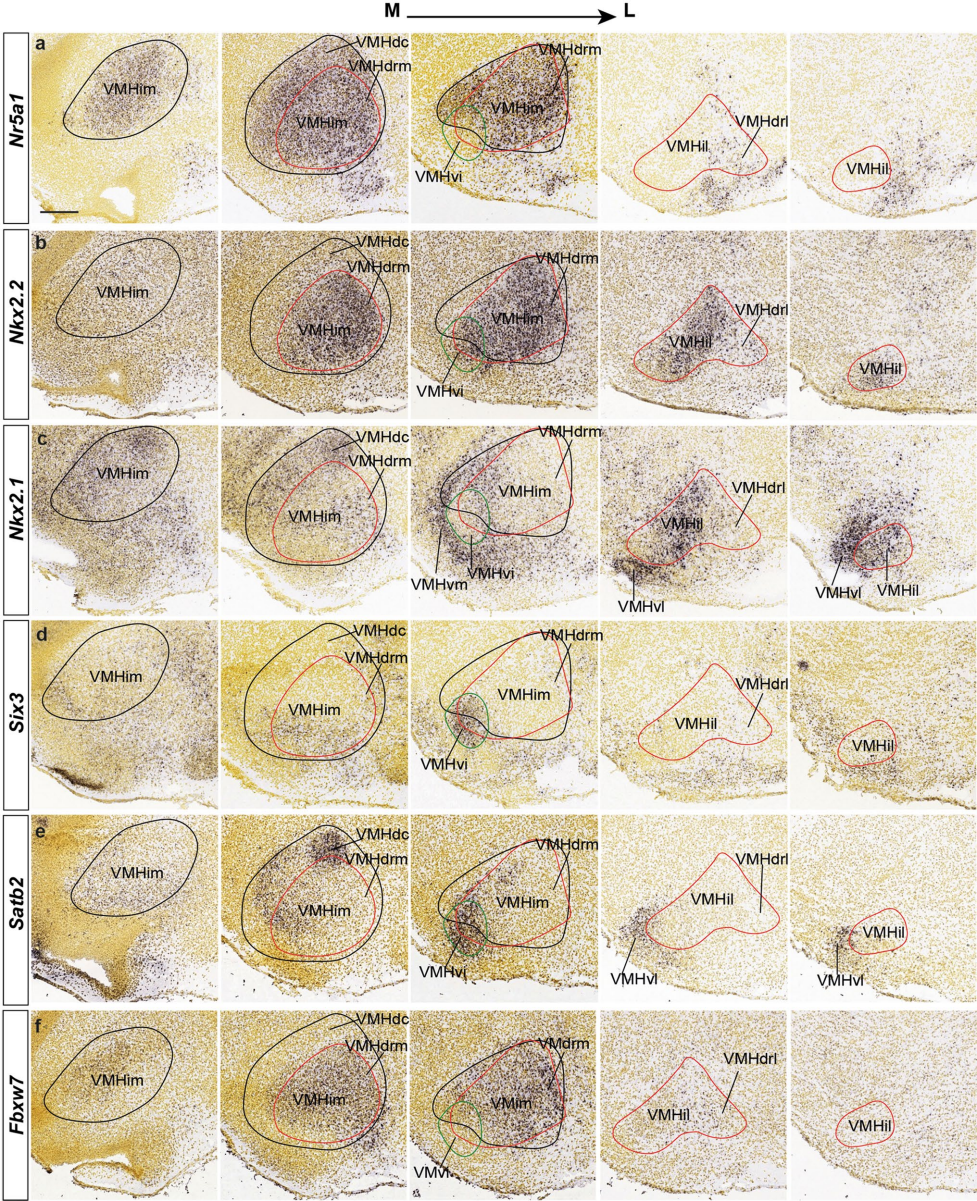

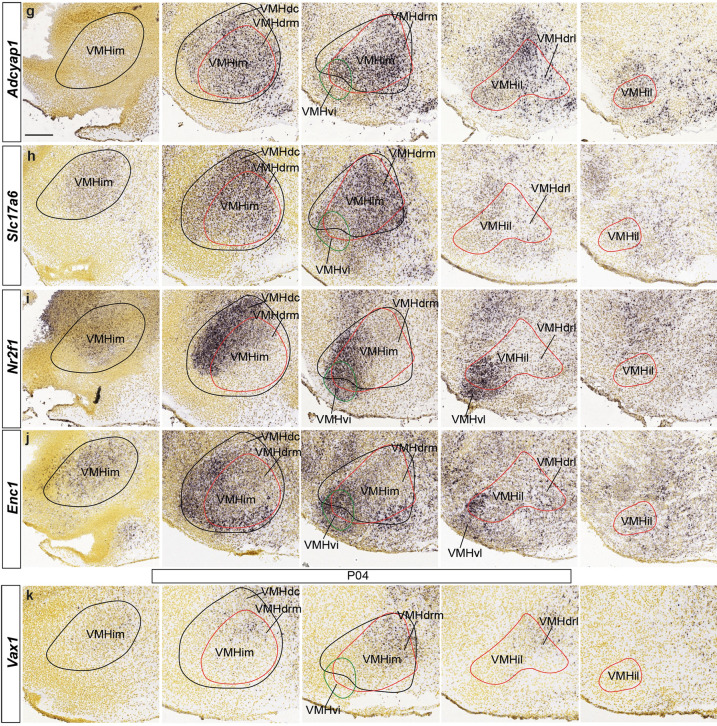
Differential dorsoventral expression of VMH markers at E18.5 or P4. This Figure (in two parts) presents for each marker (identified at left) a mediolateral series of five parallel sagittal ISH images of E18.5 (or P4, indicated) mice embryos from the Allen Developing Mouse Brain Atlas illustrating the expression pattern of the markers identified in our Tables 1, 2 across VMH. The expression patterns are compared to the outlines of *Nkx2.2* (red outline), *Nr5a1* (black outline), and *Six3* (green outline) expression. It seemed informative towards 3D assessment of relative topography to illustrate the relevant five mediolateral section levels available. We tentatively identified the VMH subdivisions described in the text (VMHdc, VMHdrm, VMHdrl, VMHim, VMHil, VMHvm, VMHvi, and VMHvl). Scale bars represent 200 µm. *VMHim* medial-intermediate VMH subnucleus, *VMHdc* dorsocaudal VMH subnucleus, *VMHdrm* dorsal-rostromedial VMH subnucleus, *VMHvi* ventral-intermediate VMH subnucleus, *VMHil* lateral-intermediate VMH subnucleus, *VMHdrl* dorsal-rostrolateral VMH subnucleus

1. The *Vax1* gene appears expressed selectively at E13.5 along the longitudinal subparaventricular alar domain (both THy and PHy; Fig. 4w, x; note this domain corresponds to the ‘hypothalamic diagonal’ of Shimogori et al. 2010 but is conceived here as strictly longitudinal; check Fig. 1); at E18.6 a *Vax1*-positive subpopulation was identified within VMHdrm/VMHdrl (Fig. 5k).
2. 4 genes -*Sema3a*, *Nkx2.2*, *Fbxw7,* and *Tcf7l2*- were selectively expressed at E13.5 at the TuD zone (implying separately either the main or subliminal parts of TuD, or encompassing both of them, as well as the corresponding TuD acroterminal portion; Fig. 4a, b, e, f, m, n). The same markers subsequently label at E18.5 mainly our dorsal VMH subdivisions (VMHdc/VMHdrm/VMHdrl) but also extend variously into the intermediate ones (VMHim/VMHil) (Fig. 5b, f).
3. Three genes -*Nr2f1*, *Lmo4*, *Satb2*- appeared expressed exclusively at the TuI domain at E13.5 or earlier (Fig. 4k, l, s, t); these labeled subsequently our intermediate and ventral VMH subdivisions at E18.5 (VMHim/il, VMHvm/vl; Tables 2; Fig. 5e, i). Two of these markers included also the VMHdc subdivision (old dorsomedial part).
4. 12 genes in Table 1 are expressed both within TuD *and* TuI domains at E13.5 (Fig. 4c, d, g, h, q, r, o, p, u, v). They can we regrouped according to the location at E18.5 of the corresponding labeled cell populations (Fig. 5a, c, g, h, j): *Nr5a1* and *Robo2* have a similar E18.5 pattern, labeling only the VMHdc, VMHdrm, and VMHim subnuclei. *Calb1* and *Slc17a6* share labeling within VMHdrm, VMHim, and VMHil at E18.5 (though *Slc17a6* signal also extends into VMHdc). Several genes -*Enc1*, *Bcl11a*, *Dner*, *Chl1*- share a strong presence at the VMHvm and VMHvl subdivisions, though their signals variously spread also into other neighboring subnuclei. The genes *Cnr1*, *Mapt*, and *Nkx2.1* share expression in the VMHdc, VMHdrm, and VMHvl, with some variable extra locations.
5. *Six3* was initially expressed at E13.5 at the acroterminal tuberal region (as well as in alar acroterminal regions; Fig. 4i, j; note this gene labels the prospective acroterminal domain already from neural plate stages onwards; Lagutin et al. 2003), with some extension into TuI. Distinct *Six3*-labeling was displayed later at E18.5 by the novel VMHvi subnucleus (Fig. 5d; note this distinct subnucleus was never described before within the classic ‘ventrolateral’ sector). These *Six3*-positive cells are interpreted to migrate from the acroterminal TuD area and/or the arcuate TuI acroterminal area, which also expresses initially *Six3*.

There clearly exist areas of overlap between most of the markers studied, indicating that most VMH subdivisions are subtly heterogeneous in molecular profile, with regional variations. This is consistent with the molecular diversity observed in transcriptomic studies (see Discussion). The VMH nevertheless shows on the whole significant partial dorsoventral, mediolateral, and rostrocaudal sorting of its molecularly distinct subpopulations. These may be tentatively classified according to their apparent positional neuroepithelial origins, tangential versus radial migration routes, and differential molecular profile. The classic schema of three VMH parts (dorsomedial, central, ventrolateral) is too simple to account for the level of heterogeneity observed (e.g., it completely misses the *Six3*-positive VMHvi subdivision as well as the dorsorostral VMH subdivisions described here and our medial–lateral distinctions; Table 3).

**Table 3:** Terminological equivalence of the main nuclei and histogenetic areas contemplated in this report according to the nomenclature of the columnar and prosomeric forebrain models

Columnar term	Abbr	Columnar histogenetic area	Prosomeric term	Nuclear Abbr	Prosomeric histogenetic area	Areal Abbr	Prosomere	Hypothalamic part	Population origin related literature
*Supramamillary nucleus medial part*	*SUMm*	*Mamillary*	Retromamillary nucleus medial part	RMM	Retromamillary area	RM	hp1	PHy	
*Supramamillary nucleus lateral part*	*SUMl*	*Mamillary*	Retromamillary nucleus lateral part	RML	Retromamillary area	RM	hp1	PHy	
*Medial mamillary nucleus*	*MM*	*Mamillary*	Medial mamillary	MM	Mamillary area	M	hp2	THy	
*Lateral mamillary nucleus*	*LM*	*Mamillary*	Lateral mamillary nucleus	LM	Mamillary area	M	hp2	THy	
*Posterior hypothalamus, hypothalamic part*	*PHr*	*Mamillary*	Periretromamillary band	PRM	Periretromamillary area	PRM	hp1	PHy	
*Posterior hypothalamus, diencephalic part*	*PHc*	*Prerubral tegmentum*	Prerubral tegmentum	p3Tg + p2Tg	p3 and p2 basal plate	p3B, p2B	p3 + p2	Dienc	Puelles et al. (2012)
*Tuberomamillary nucleus + Dorsal premamillary nucleus*	*TM*	*Mamillary*	Perimamillary band + Dorsal premamillary nucleus	PM DPM	Perimamillary area Retrotuberal ventral area and Tuberal ventral area	PM	hp2	THy	
*Tuberomamillary histaminergic neurons*	*TM*	*Mamillary*	Ventral retrotuberal and tuberal areas	RTuV + TuV	Ventral retrotuberal and tuberal areas	RTuV + TuV	hp1 + hp2	PHy + THy	
*Ventral premamillary nucleus*	*PMv*	*Mamillary*	Ventral premamillary nucleus, core	VPMc	Migrated tangentially from retromamillary área (located TuI)	RM	hp2	THy	López-González et al. (2021)
			Ventral premamillary nucleus, shell	VPMsh					
*Dorsomedial hypothalamic nucleus, anterior part*	*DMHa*	*Tuberal*	Dorsomedial hypothalamic nucleus, peduncular part (core/shell)	DMHc/s-P	Intermediate retrotuberal area	RTuI	hp1	PHy	Díaz et al. (2015)
*Dorsomedial hypothalamic nucleus, posterior/ventral part*	*DMHp/v*	*Tuberal*	Dorsomedial hypothalamic nucleus terminal part (core/shell)	DMHc-P	Intermediate tuberal area	TuI	Hp2	THy	Díaz et al. (2015)
*Subthalamic nucleus*	*STN*	*Mamillary*	Subthalamic nucleus	STh	Migrated dorsalward from retromamillary area	RM	hp1	PHy	Skidmore et al. (2008)
*Parasubthalamic nucleus*	*PSTN*	*Mamillary*	Parasubthalamic nucleus	PSTh	Mifrated dorsalward from retromamillary area	RM	hp1	PHy	
*Arcuate hypothalamic nucleus*	*ARH*	*Tuberal*	Arcuate nucleus	Arc	Intermediate tuberal área (acroterminal part)	TuI	hp2	THy (acroterminal)	
*Ventromedial hypothalamic nucleus, dorsomedial part*	*VMHdm*	*Tuberal*	Ventromedial hypothalamic nucleus, dorsocaudal part	VMHdc	Intermediate tuberal area	TuI	hp2	THy	Present results
*Ventromedial hypothalamic nucleus, anterior part*	*VMHa*	*Tuberal*	Ventromedial hypothalamic nucleus, rostromedial dorsal part	VMHdrm	Dorsal tuberal área + Alar subparaventricular area	TuD + SPa	hp2	THy	Present results
			Ventromedial hypothalamic nucleus rostrolateral dorsal part	VMHdrl					
*Ventromedial hypothalamic nucleus, central part (core)*	*VMHc*	*Tuberal*	Ventromedial hypothalamic nucleus, intermedio-medial part	VMHim	Dorsal tuberal area	TuD	hp2	THy	Present results
			Ventromedial hypothalamic nucleus, intermedio-lateral part	VMHil					
*Ventromedial hypothalamic nucleus, ventrolateral part*	*VMHvl*	*Tuberal*	Ventromedial hypothalamic nucleus, ventromedial part	VMHvm	Intermediate tuberal area	TuI	hp2	THy	Present results
			Ventromedial hypothalamic nucleus, ventro-intermediate part	VMHvi					
			Ventromedial hypothalamic nucleus, ventrolateral part	VMHvl					

For the columnar terms (italic) we used the terminology, schemata, and table of Hahn et al (2019), whereas for the prosomeric terms (roman) we used Puelles et al (2012) and VMH subdivision concepts introduced in the present report as reference

### Development of ventromedial nucleus subpopulations marked with selected genes

To analyze in more detail the variant development of VMH subpopulations according to their progenitor origins, we selected for follow-up a group of genes expressed in distinct progenitor areas at E13.5 whose derivatives constitute characteristic VMH subpopulations at E18.5: *Vax1*, *Nkx2.2*, *Nr5a1*, *Satb2, Tcfl2, Sox14, Nr2f1, Nkx2.1,* and *Six3*. Origins associated to the TuI progenitor area—cases of *Satb2, Nr2f1,* and *Nkx2.1*- imply a priori a radial migration pattern, whereas origins associated to the overlying alar TSPa or to the TuD—cases of *Vax1*, *Nkx2.2*, *Nr5a, Tcfl2,* and *Sox14*- suggest tangential dorsoventral displacements. At least in the case of *Six3* it is possible to study separately a restricted acroterminal origin, which would involve a priori a rostral source and a caudally oriented tangential displacement.

#### Vax1

**Fig. 6:**
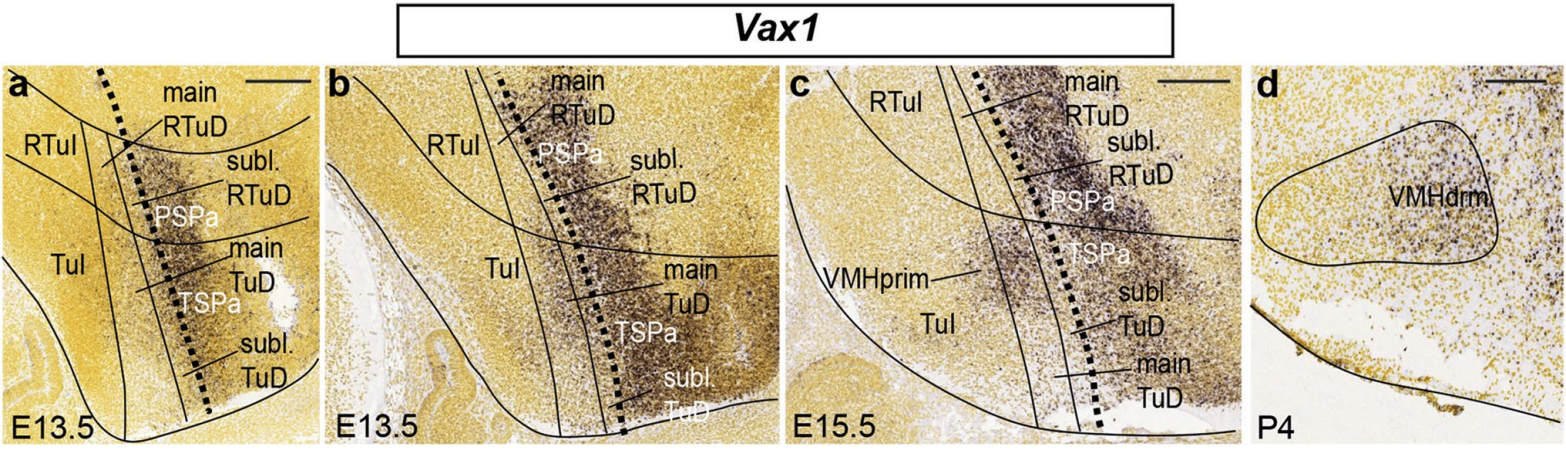
Apparent alar origin of Vax1-expressing VMH cells. **a**, **b** Sagittal images illustrating two *Vax1* ISH-reacted sections in E13.5 mice from the Allen Developing Mouse Brain Atlas. The black dashed line identifies the alar-basal boundary (alar to the right; basal to the left, as in Fig. 1**b**). The TuI, main TuD and subliminal TuD progenitor domains are delimited by solid lines. At **b** there appears *Vax1* signal penetrating the underlying TuD area. **c** At E15.5, the area showing displaced *Vax1* labeling extends ventralwards into a dorsal part of the VMH primordium. **d** The final VMH configuration displays *Vax1* transcripts mainly at its VMHdrm/VMHdrl subdivisions (shown at P4). Scale bars represent 200 µm. *RTuI* retrotuberal intermediate area, *TuI* tuberal intermediate area, main or subl. RTuD, main or subliminal retrotuberal dorsal area; main or subl. *TuD* main or subliminal tuberal dorsal area, *PSPa* peduncular subparaventricular area, *TSPa* tuberal subparaventricular area, *primVMH* primordium of the ventromedial hypothalamic nucleus, *VMHdrm* dorsal-rostromedial VMH subnucleus

*Vax1* is a typical early selective marker of the alar subparaventricular area, which extends over the terminal and peduncular hypothalamic domains (PSPa and TSPa) and includes at its ventral rim the liminal alar *Nkx2.2*-positive zone (Puelles et al. 2012; in blue in Fig. 1a, b). Initially the expression of *Vax1* ends strictly at the alar-basal boundary. *Vax1*-labeled cells start to move into the subjacent basal tuberal region at E13.5 (Fig. 6a, b). Two days later, this marker delineates rostrodorsal parts of the VMH primordium (Fig. 6c, d; note this result changes the classic simpler concept of the dorsal region of VMH, contemplating only the caudally placed ‘dorsomedial VMH part’; obviously, our ‘dorsorostral’ descriptor refers to the prosomeric axis and columnar authors may want to use a different descriptor). At postnatal stages the tuberal expression of *Vax1* coincides mainly with the VMHdrm/VMHdrl subnucleus with some dispersion into VMHim/VMHil (Figs. 5k; 6d).

#### Nkx2.2

**Fig. 7:**
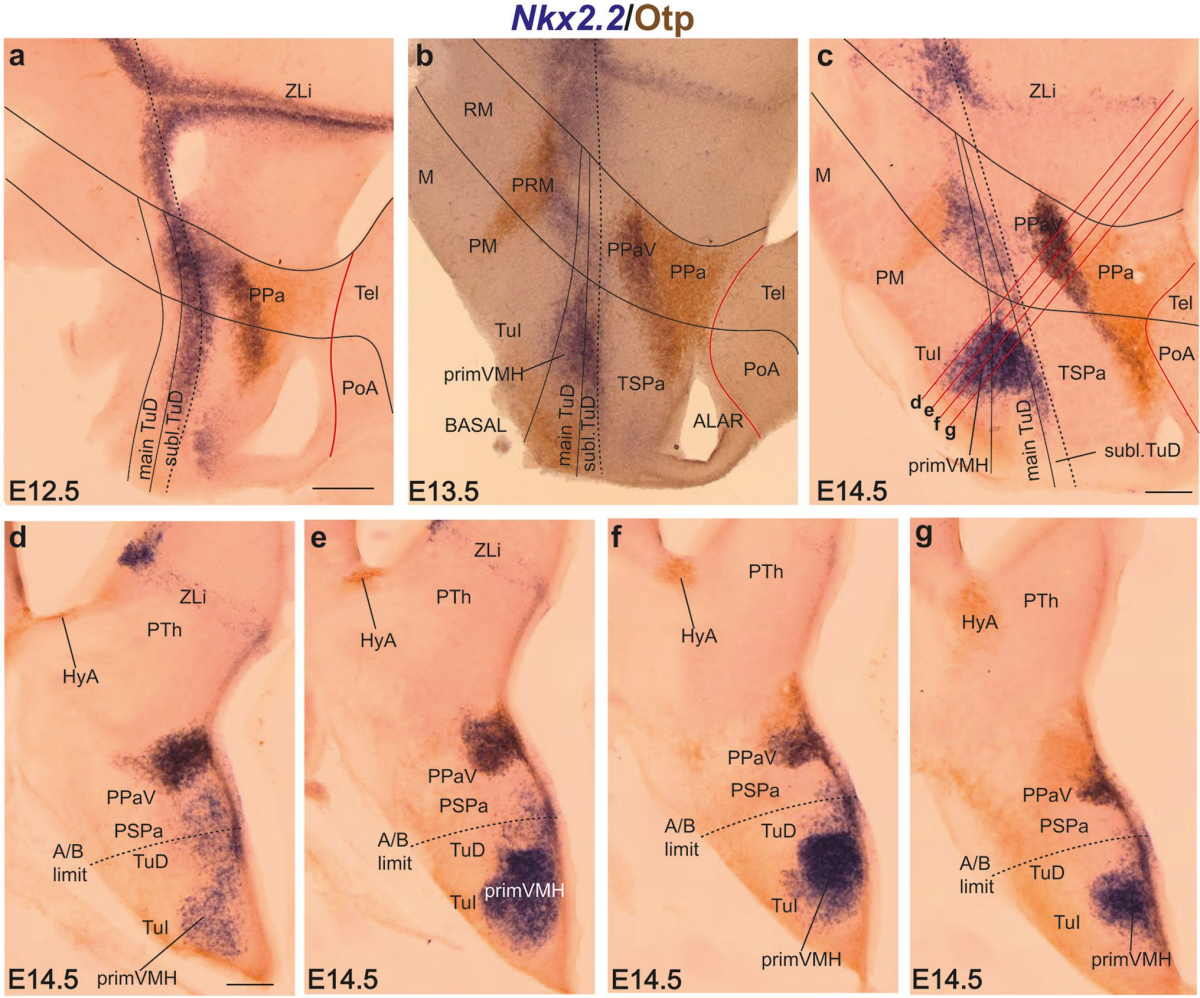
Evolution of *Nkx2.2* and Otp signals relative to the emergence of the VMH primordium between E12.5 and E14.5. All panels show double *Nkx2.2* ISH/Otp IHC in sagittal **a**–**c** or oblique **d**–**g** sections from E12.5, E13.5 and E14.5 embryos (levels of section in **d**–**g** indicated in **c**). The dash black line always marks the alar-basal boundary and roughly parallel solid black lines delimit the subliminal and main TuD areas in (**a**–**c**). The curved red line in **a**–**c** identifies the hypotalamo-telencephalic boundary dorsal to the Otp-positive paraventricular area (PPa; brown). **b** The blue *Nkx2.2* signal previously related only to the subliminal TuD (**a**) now clearly starts to extend ventralwards into the TuI territory at E13.5 (primVMH). **c** This process progresses considerably by E14.5 (primVMH). Note that a less important ventral expansion of *Nkx2.2* cells is observed likewise at the RTuD domain, which comes to contact the Otp-positive periretromamillary band (PRM). **d**–**g** The original early *Nkx2.2*-positive subliminal band (blue) shows a dorsalward migration into the ventral part of the Otp-positive alar paraventricular area (PPaV; **a**–**c**; **d**–**g**), and this process later expands rostralwards into THy, in parallel to the ventralward migratory phenomena leading to the tuberal VMH; the intercalated subparaventricular alar area (PSPa) is crossed subventricularly by the dorsally migrating cells, but essentially remains unlabelled, excepting some liminal expression (PPa; TSPa; primVMH; **a**–**c**). Our oblique sections through this area intersect the VMH primordium and the PPaV at four levels, providing images consistent with a migratory interpretation. Note that the VMH *Nkx2.2* cell population does not represent the entire VMH population. Scale bars represent 200 µm. *ZLi* zona limitans, *Main or subl. TuD* main or subliminal tuberal dorsal area, *PPa* peduncular paraventricular area, *PPaV* ventral peduncular paraventricular nucleus, *Tel* telencephalon, *PoA* preoptic area, *RM* retromamillary area, *M* mamillary area, *PRM* periretromamillary area, *PM* perimamillary area, *TuI* tuberal intermediate area, *primVMH* primordium of the ventromedial hypothalamic nucleus, *PTh* prethalamus, *HyA* hypothalamo-amygdalar corridor, *PSPa*, peduncular subparaventricular area, *A/B limit* alar/basal limit

*Nkx2.2* is well known as an early longitudinal marker of the alar-basal boundary along the whole forebrain tagma (midbrain, diencephalon, and hypothalamus; note this is a basic token of the updated *extended forebrain* concept held within the prosomeric model; Puelles et al. 2012; Puelles and Rubenstein 2015; Puelles 2018; Amat et al. 2022). This lineal signal also appears bordering the spike of *Shh* expression that marks the transverse alar *zona limitans intrathalamica* (alar p3/p2 boundary), also known as the *mid-diencephalic organizer* (Puelles and Martínez 2013). This gene (as well as others such as *Nkx2.9, Ptc*, etc.) is apparently selectively upregulated by particularly high local concentrations of SHH signal diffusing dorsalward from the underlying floor and basal plate *Shh* expression domain or from the related ZLI core domain which obeys a different enhancer (Briscoe et al. 1999; Puelles and Martínez 2013; Nishi et al. 2015; Andreu-Cervera et al. 2018). In the hypothalamus, *Nkx2.2* signal appears early on as a thin longitudinal band that *overlaps* the lineal boundary between the alar and basal plates and stops as it reaches the acroterminal border (Fig. 7a). The mixed alar-basal expression led to the concepts of alar *liminal* and basal *subliminal* subdivisions of the *Nkx2.2* band (Puelles et al. 2012; ‘liminal’ refers to the classic notion of *limen lamina alaris*, or rim of the alar plate; see red and blue bands in Fig. 1a, b). The subliminal subdomain thus forms the upper rim of the TuD/RTuD basal tuberal hypothalamic progenitor area, whereas the liminal area is a subdomain at the ventral rim of the subparaventricular hypothalamic area across THy and PHy. We will refer to the *non-subliminal* larger ventral part of the TuD/RTuD area as ‘the main TuD/RTu’ (Fig. 1b). These distinctions are not contemplated in the columnar tradition (Table 3).

**Fig. 8:**
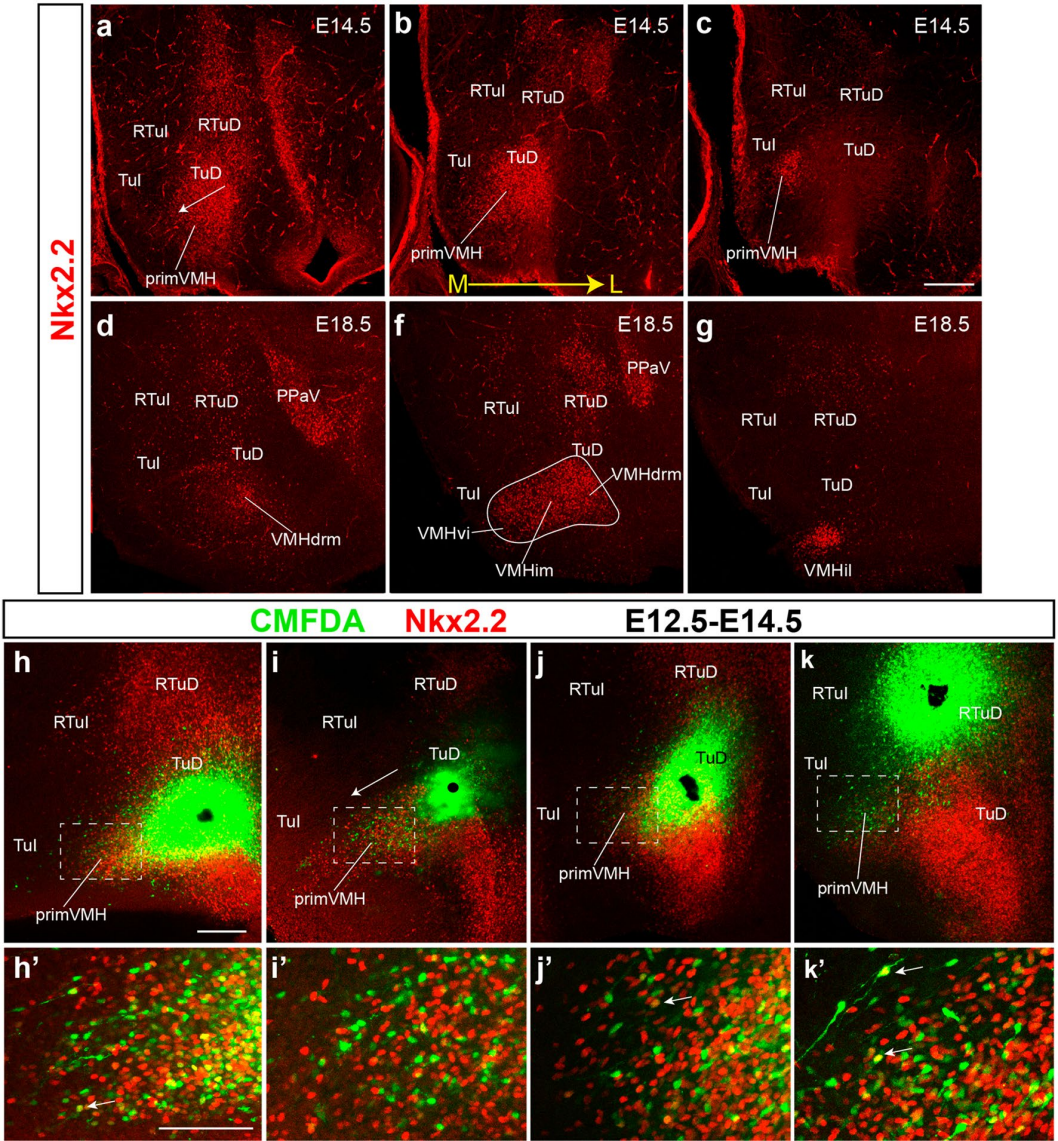
A Nkx2.2-expressing population partially connected with the TuD reaches the TuI domain to form part of the VMH primordium. **a**–**g** Sagittal sections in medio–lateral order comparing the VMH primordium labeled with anti-Nkx2.2 antibody in red fluorescence at migratory stage E14.5 (**a**–**c**) and postmigratory stage E18.5 (**d**–**g**). The approximate contour of the whole VMH nucleus was traced with a white line in **f**. At E18.5 the specific subdivisions of the VMH that are invaded by this cell type can be identified tentatively (VMHdrm, VMHim, VMHil, VMHvi). **h**–**k** Representative sagittal images of our tracing experiments on organotypic cultures from E12.5 mice (48 h culture conditions, E12.5-E14.5), marked with the anti-Nkx2.2 antibody (red), and illustrating ventralwards cell migration when a CMFDA particle (black) was placed at the upper part of TuI (**h**), the TuD (**i**, **j**), or the RTuD-TuD boundary (**k**). (**h**’–**k**’) Higher magnification images from the dashed square areas in (**h**–**k**). White arrows in **h**’,**j**’,**k**’ indicate cells that correspond to double-labeled CMFDA-Nkx2.2 elements. The white arrows in **a**, **i** indicate the direction of Nkx2.2 or CMFDA label extension at higher magnification in the respective framed areas. Scale bars in **c**, **h** represent 200 µm. Scale bar in **h’** represents 100 µm. *RTuI* retrotuberal intermediate area, *TuI* tuberal intermediate area, *RTuD* retrotuberal dorsal area, *TuD* tuberal dorsal area, *primVMH* primordium of the ventromedial hypothalamic nucleus, *PPaV* ventral peduncular paraventricular nucleus, *VMHdrm* dorsal-rostromedial subnucleus of the ventromedial hypothalamic nucleus, *VMHim* medial-intermediate VMH subnucleus, *VMHvi* ventral-intermediate VMH subnucleus, *VMHil* lateral-intermediate VMH subnucleus

Results on zebrafish embryos indicate that the earliest appearance of *Nkx2.2* signal occurs at neural plate stages (Hauptmann and Gerster 2000; Hauptmann et al. 2002). In E12.5 mouse embryos *Nkx2.2* is already expressed longitudinally along the alar-basal boundary of the midbrain, diencephalon, and hypothalamus (Fig. 7a). At E13.5, *Nkx2.2-*expressing cells start to migrate ventralwards (mainly within THy) and the corresponding signal begins to protrude ventralwards under the terminal subliminal band strictly expanding within the basal tuberal region; this represents the first sign of the migration of *Nkx2.2* cells into the VMH primordium (primVMH; Fig. 7b). This process is clearly more advanced at E14.5 (primVMH; Figs. 7b, c, 8a–c). Cross-sections at E14.5 illustrate that *Nkx2.2* signal is present at the ventricular and mantle zones of the *subliminal* and *main* parts of the TuD, as well as in the underlying VMH nuclear primordium (only mantle zone), while the TuI ventricular zone deep to VMH remains negative (primVMH; Fig. 7d–g). At E18.5 there is already a definitive distribution pattern of *Nkx2.2* signal within VMH (Fig. 8d–g). The dorsoventral tubero-tuberal VMH migration stops within TuI at some distance of the perimamillary band, leaving a substantial space for the terminal part of the DMH nucleus, as well as for the separately tangentially migrated VPM nucleus (López-González et al. 2021). Interestingly, at E13.5-E14.5 there is also a previously unidentified shorter parallel dorsoventral *Nkx2.2* migration coming out of the *peduncular retrotuberal* subliminal band that extends ventralwards into RTu, where it stops in contact with the *Otp*-positive periretromamillary band; this parallel migration is distinctly separated from the VMH and may contribute *Nkx2.2*-expressing neurons to the dorsomedial nucleus (Fig. 7b, c).

We performed some in vitro fluorescent labeling experiments on organotypic cultures of embryonic hypothalamus to visualize the ventralward tubero-tuberal migration into VMH originated from the primary *Nkx2.2*-expressing band across the alar-basal boundary of THy. A small particle of CMFDA (see Methods) was placed at the TuD in E12.5 explants and further analysis was done at E14.5, at which stage the VMH is already fairly well formed, though the migration is not complete yet. All explants were treated for Nkx2.2 immunofluorescence to check the position of the CMFDA particle, and in all cases (*n* = 6) a stream of CMFDA-labeled cells overlapped with the Nkx2.2-positive cells advancing into TuI, showing a comparable disposition (*n* = 4 in Fig. 8h–k). Some Nkx2.2/CMFDA-positive migrating cells were observed relative to the labeling sites placed across the width of the Nkx2.2-positive band (white arrows in Fig. 8h’–k’). This reveals that some dorsoventrally migrating cells do not express Nkx2.2, which possibly includes the *Nr5a1*-expressing cells (see next section) and/or passing *Vax1* cells coming from the overlying alar subparaventricular area (previous section). In one experiment in which the CMFDA particle was placed at the TuD/RTuD limit where it crosses the orthogonal THy/PHy border migrated CMFDA-Nkx2.2 double-labeled cells were found also within the VMH primordium (Fig. 8k, k’). This result is consistent with descriptive images in which Nkx2.2-expressing cells seem to originate throughout the TuD/RTuD boundary, appearing in transient continuity with the VMH primordium (Figs. 7b, 8k).

#### Nr5a1

A comparison of the relatively complementary expression of *Nkx2.2* and *Nr5a1* within VMH illustrates the basic organization we propose for the ventromedial primordium. *Nr5a1* (previously known as steroidogenic factor 1, *SF1*), has been widely analyzed developmentally and functionally, and is sometimes considered a selective transient marker of the entire VMH nucleus (Ikeda et al. 1995; Shinoda et al. 1995; Dellovade et al. 2000; Tran et al. 2003; Davis et al. 2004; Dhillon et al. 2006; Kim et al. 2011, 2019; Büdefeld et al. 2012; Cheung et al. 2013; see Discussion). However, this contrasts with our present data, given that most *Nkx2.2*-positive VMH cells seem to be *Nr5a1* negative. *Nkx2.2* has received relatively less attention so far as a VMH marker (Kurrasch et al. 2007; Puelles et al. 2012; Corman et al. 2018).

**Fig. 9:**
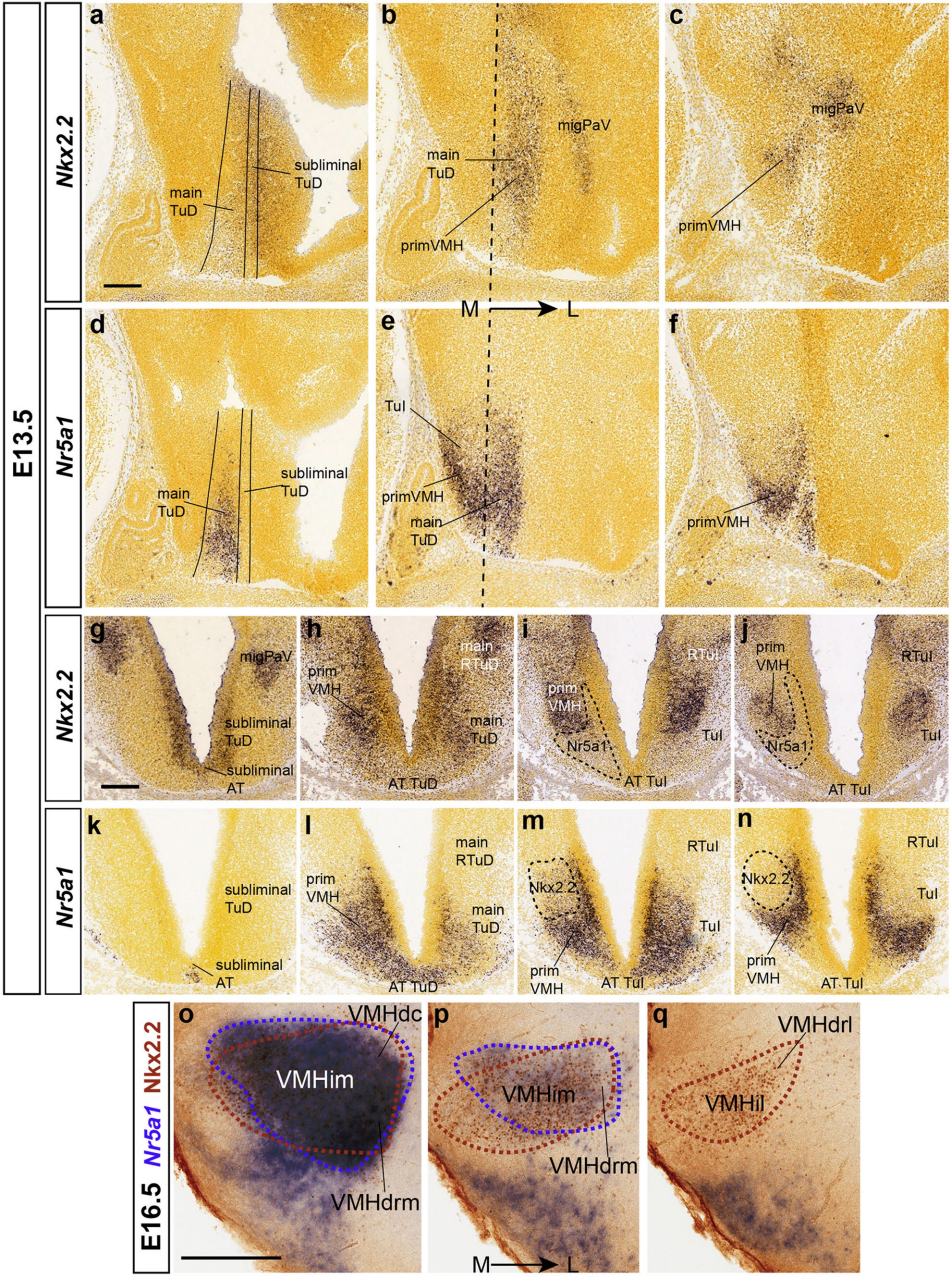
*Nkx2.2* and *Nr5a1* reveal different migrated VMH populations. **a**–**n** *Nkx2.2* and *Nr5a1* ISH reactions are compared in mediolateral sagittal (**a**–**f**) and dorsoventral horizontal (**g**–**n**) sections of E13.5 mouse embryos from the Allen Developing Mouse Brain Atlas (horizontal relative to the prosomeric axis), illustrating the postulated zones of origin of the populations expressing selectively one or the other of these markers and their topographic relationship with the ventrally displaced and expanding VMH primordium, where both populations remain spatially distinct. Sections (**b**, **c**, **g**, **h**) also show *Nkx2.2*-expressing cells that migrate dorsalward into peduncular and terminal parts of the ventral paraventricular subarea (migPaV). The black dash line in **b**, **e** marks the prosomeric horizontal section plane illustrated at various levels in **g**–**n**. The section in **g** is slightly oblique and shows *at the left side* alar (peduncular and terminal) PSPa and TSPa regions bordering ventrally the liminal ventricular zone, whereas *at the right side* we see the transition into the underlying basal subliminal ventricular zone; note none of these sides shows significant mantle layer labeling of VMH nature, which only starts to appear in the underlying sections **h**–**j** and **l**–**n**, always restricted to the terminal hypothalamus in what respects the VMH primordium. Note in **h**–**j** and **l**–**n** that at E13.5 the two cell types intermix somewhat at dorsal levels through VMH **h**, **l**, whereas they stay separate more ventrally **j**–**n**). **o**–**q** Combined blue *Nr5a1* ISH and brown Nkx2.2 immunohistochemical sagittal images at E16.5 show that intermixing increases significantly by this stage. A blue dash contour was traced around the *Nr5a1*-positive VMH population, and a brown dash contour surrounds the Nkx2.2-positive VMH population, allowing this comparison. Scale bars represent 200 µm. *TuD* tuberal dorsal area, *migPaV* migration of the ventral paraventricular cells, *primVMH* primordium of the ventromedial hypothalamic nucleus, *AT* acroterminal area, *RTuI*, retrotuberal intermediate area, *TuI* tuberal intermediate area, *RTuD* retrotuberal dorsal area, *VMHdc* dorsocaudal VMH subnucleus, *VMHim* medial-intermediate VMH subnucleus, *VMHdrm* dorsal-rostromedial VMH subnucleus, *VMHil* lateral-intermediate VMH subnucleus, *VMHdrl* dorsal-rostrolateral VMH subnucleus

In contrast to *Nkx2.2*, the gene *Nr5a1* is excluded early on from the narrow *subliminal* TuD band but is strongly expressed at the underlying intrinsically *Nkx2.2* negative *main* TuD area (though migrating Nkx2.2 cells pass through it), including the associated acroterminal TuD domain, fully devoid of *Nkx2.2* signal (Fig. 9a, d, g, k). At E13.5, *Nr5a1*-positive cells are present at the deepest stratum of the local (TuD) mantle, extending into TuI (Fig. 9d–f, k–n). These cells thereafter invade the deep region of the underlying VMH primordium (Fig. 9k–n). The migrating deep stream of *Nr5a1* cells thus seems covered by the more superficially migrating separate stream of *Nkx2.2* cells (Fig. 9a–f, h–j, l–n). The migration of *Nr5a1* cells originating at the *Nr5a1*-positive acroterminal TuI area is oriented ventrocaudally, apparently incorporating into the main TuD migration into VMH. Once the *Nr5a1* cells reach the deep part of VMH they spread out radially and tangentially within the nucleus (Fig. 9l–n). At E16.5 *Nr5a1* cells are visible mainly medially at the VMHdrm, and VMHim subdivisions, with a medio-laterally decreasing density gradient partially overlapping with *Nkx2.2* cells in an inverse lateromedial gradiental distribution. At this stage we found intermixed *Nr5a1* and *Nkx2.2* populations at the VMHdrm and VMHim, whereas *Nr5a1* cells are absent at the VMHil subnucleus where *Nkx2.2* cells are massively present (Fig. 9o–q).

#### Tcf7l2/Satb2/Sox14

**Fig. 10:**
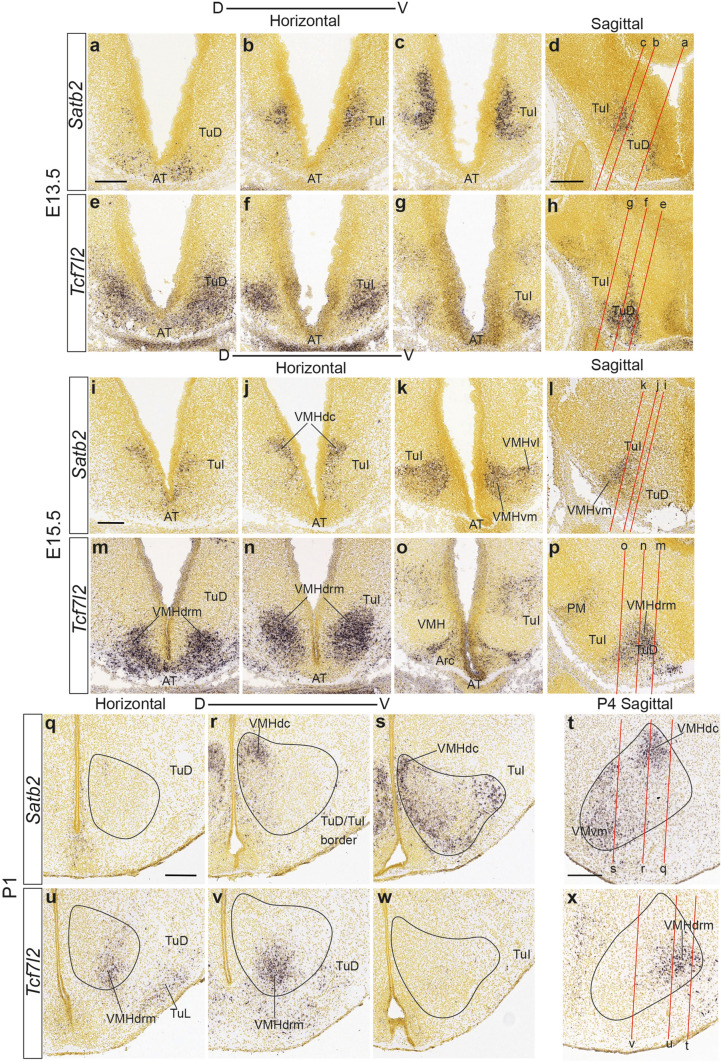
*Comparisons of Satb2* and *Tcf7l2* patterns reveal a rostrocaudal subdivision in the dorsal sector of VMH. *Satb2* and *Tcf7l2* ISH images from the Allen Developing Mouse Brain Atlas are shown in either prosomeric horizontal sections at three dorsoventral levels at E13.5 (**a**–**c**, **e**–**g**), E15.5 (**i**–**k**, **m**–**o)**, and P1 (**q**–**s**, **u**–**w**) or in corresponding sagittal sections (**d**, **h**, **l**, **p**, **t**, **x**). The *Satb2* marker is expressed initially only within TuI, where a dense periventricular stratum of positive cells is visible at E13.5 (**a**, **b**; note additional VMH-unrelated expression appears also at the acroterminal TuD area in **a**, **d**). At E15.5 the dorsalmost VMH *Satb2 *cells concentrate at the VMHdc subdivision (**i**, **l**), while other cells of this type have invaded massively the ventral parts of the VMH primordium (VMHvm; VMHvl; **j**, **k**). At P1 the *Satb2* subpopulation persists majoritarily at the VMHdc and the ventral complex (**q**–**t**). In contrast, the *Tcf712* marker initially appears expressed selectively at the acroterminal TuD area (**e**–**h**); later, *Tcf7l2* cells migrate ventralwards into the rostral part of the VMH primordium (**m**–**p**), invading mainly the VMHdrm subdivision, while other acroterminal TuD cells seem to invade the TuL nucleus (**u**–**x**) (**e**–**h** for E13.5; **m**–**p** for E15.5; **q**–**x** for P1). Scale bars represent 200 µm. *AT* acroterminal domain, *TuD* tuberal dorsal area, *TuI* tuberal intermediate area, *VMHdc* dorsocaudal VMH subnucleus, *VMHvl* ventral-lateral VMH subnucleus, *VMHvm* ventral-medial VMH subnucleus, *VMHdrm* dorsal-rostromedial VMH subnucleus, *VMH* ventromedial hypothalamic nucleus, *PM* perimamillary area, *TuL* tuberal lateral nucleus

**Fig. 11:**
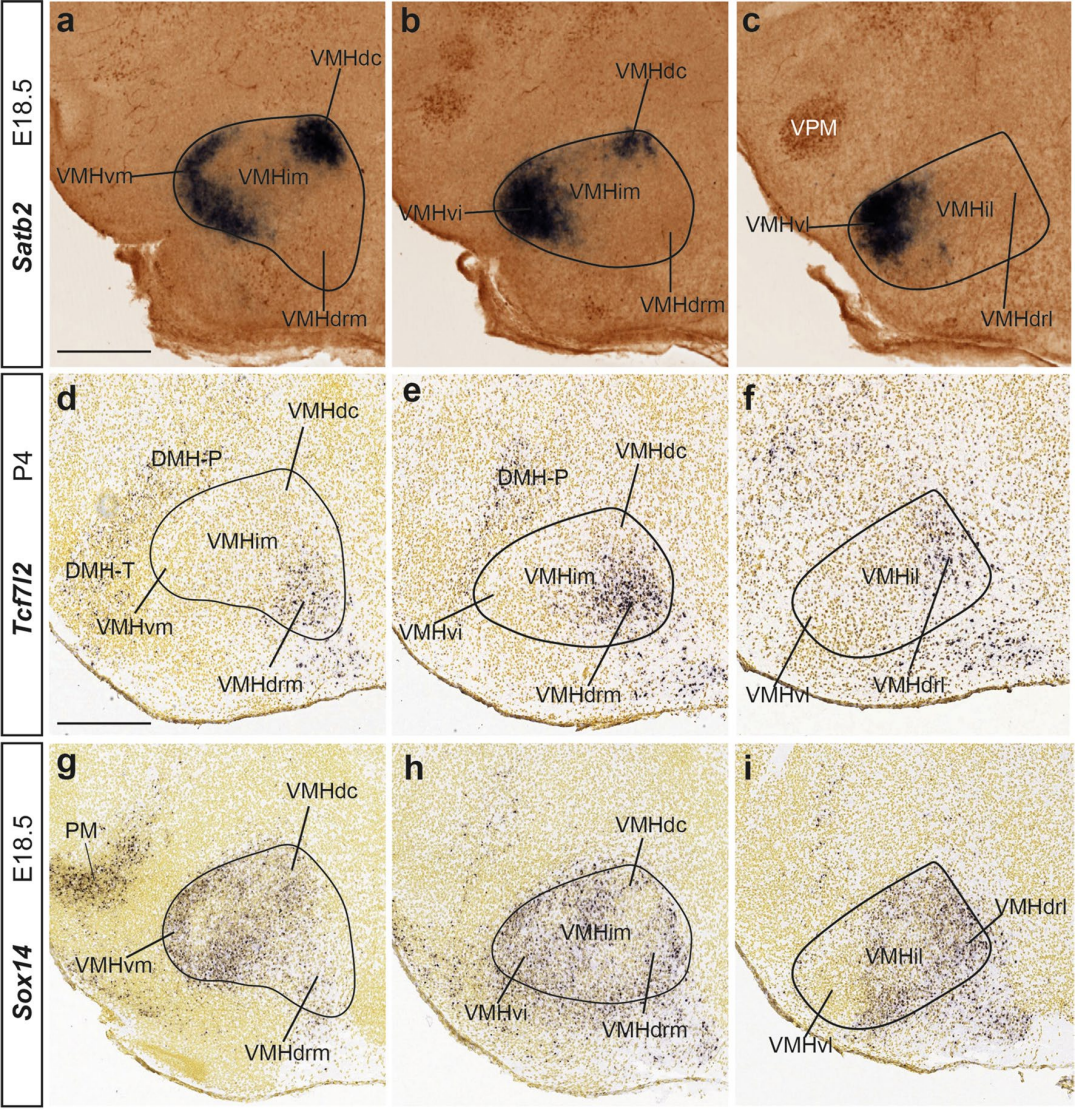
Differentially labeled populations of the VMH at late developmental stages. *Satb2* **a**–**c**, *Tcf712* **d**–**f***,* and *Sox14* **g**–**i** ISH mediolateral sagittal sections of E18.5 or P4 mice. These tree markers are differentially expressed in diverse VMH subnuclei: *Satb2* labels distinctly the VMHdc subunit and the ventral area (VMHvm, VMHvi and VMHvl), *Tcf7l2* is selective for the VMHdrm-VMHdrl subdomains, and *Sox14* seems to coincide with *Satb2* at deep and intermediate sagittal sections (signal at VMHvm; **g**, **h**), but differs drastically at lateral levels **i**; comparison with *Tcf7l2* shows a complementary pattern at medial and intermediate levels (VMHdrm; **g**, **h**) and some coincidences at lateral levels (VMHdrl, VMHil, VMHvl). Scale bars represent 200 µm. *VMHvm* ventral-medial VMH subnucleus, *VMHim* medial-intermediate VMH subnucleus, *VMHdc* dorsocaudal VMH subnucleus, *VMHdrm* dorsal-rostromedial VMH subnucleus, *VMHvi* ventral-intermediate VMH subnucleus, *VPM* ventral premamillary nucleus, *VMHvl* ventral-lateral VMH subnucleus, *VMHil* lateral-intermediate VMH subnucleus, *VMHdrl* dorsal-rostrolateral VMH subnucleus, *DMH-P* peduncular part of the dorsomedial hypothalamic nucleus, *DMH-T* terminal part of the dorsomedial hypothalamic nucleus, *VMHvm* ventral-medial VMH subnucleus

At E13.5 the *Tcf7l2* gene labels the TuD acroterminal mantle, extending somewhat into the neighboring rostral TuI. This *Tcf7l2*-TuI pattern is partially complementary to that of *Satb2*, whose expression occurs mainly at the periventricular stratum of TuI (Fig. 10a–h). At E15.5, the primordium of the VMHdrm subnucleus is selectively marked by *Tcf7l2*, whereas the VMHdc primordium and the ventral parts of VMH are marked with *Satb2* (Fig. 10i–p). At late developmental stages, the rounded VMHdrm is perfectly delineated by *Tcf7l2*, as is the VMHdc portion by *Satb2* (Figs. 10q–x, 11a–f). The latter gene also marks the three VMHvm, VMHvi and VMHvl divisions, some of which are also recognizable with *Sox14*, another TuI-TuD early marker, which appears expressed at the VMHdrl, but not at the VMHdrm companion, and similarly at the VMHvm, but not at the VMHvl (Fig. 11g–i).

#### Nr2f1

**Fig. 12:**
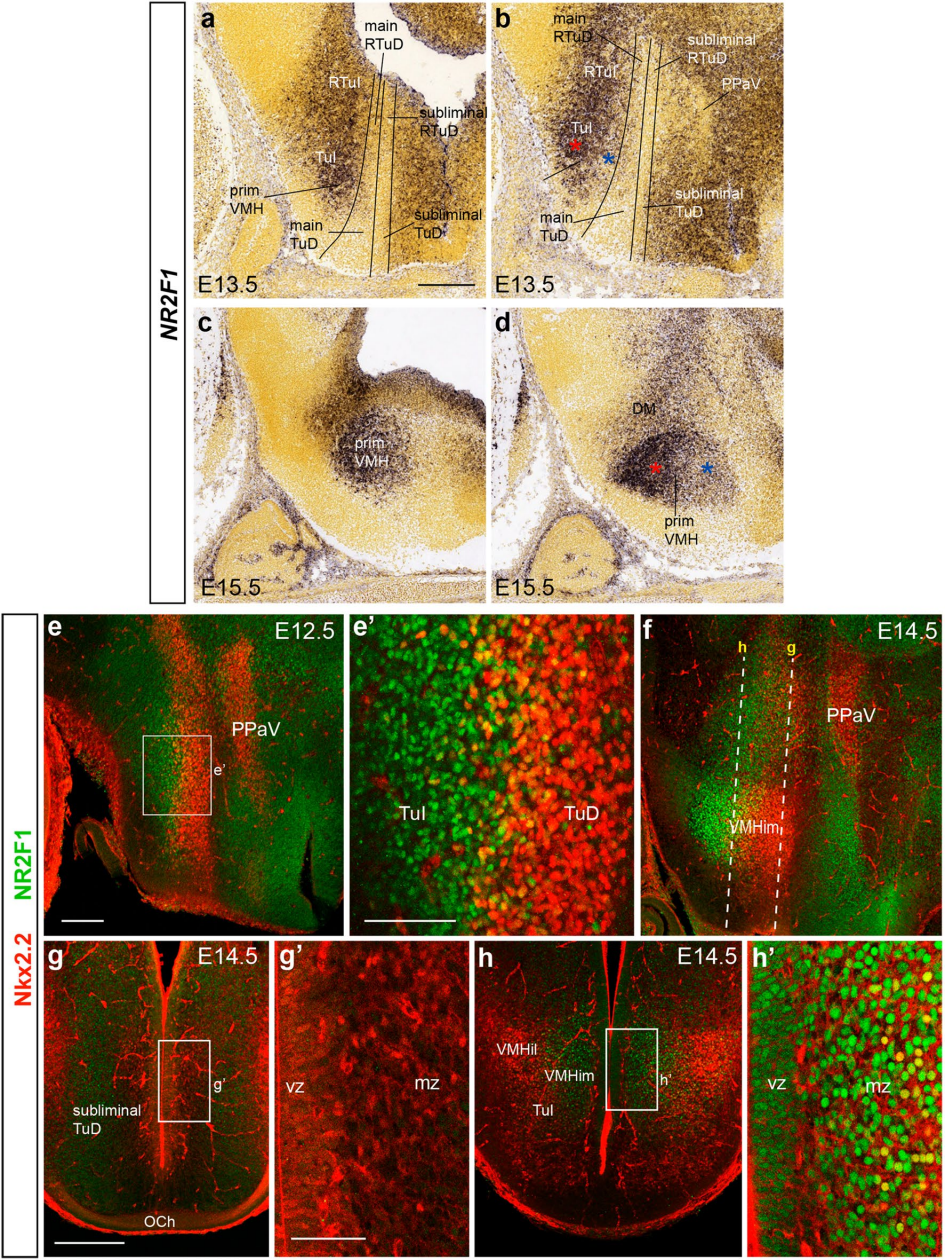
*Nr2f1* expression illustrates a significant local TuI contribution to the VMH primordium. **a**–**d** *Nr2f1* ISH sagittal images at E13.5 and E15.5 from the Allen Developing Mouse Brain Atlas. Solid lines in **a**, **b** delimit the longitudinal (subliminal) TuD/RTuD, (main) TuD/RTuD and TuI/RTuI progenitor basal domains. Red asterisks in **b**, **d** show a strong *Nr2f1* signal at the ventral part of VMH primordium and its more mature derivative. Blue asterisks in **b**, **d** identify dorsal areas of relatively weaker *Nr2f1* signal at the VMH primordium. (**e**–**h**’) Double Nkx2.2 (red) and Nr2f1 (green) immunofluorescence in sagittal (**e**–**f**) and horizontal (**g**–**h**’) sections of E12.5 and E14.5 embryos. The dash lines in **f** indicate the horizontal section planes at **g** and **h**. At TuD/RTuD levels (just dorsal to VMH) the ventricular zone and adjacent mantle zone are Nkx2.2-positive (**vz**; **mz**; **g**–**g**’), whereas more ventrally through the VMH primordium (within TuI) the ventricular zone is only Nr2f1-positive and the mantle zone has a mixture of green cells (particularly at deep levels), with red and yellow cells (**vz**; **mz**; **h**–**h**’). Scale bars in **a**, **e**, **g** represent 200 µm. Scale bar in **g**’ represents 100 µm. Scale bar in **h**’ represents 50 µm. *primVMH* primordium of the ventromedial hypothalamic nucleus, *RTuI* retrotuberal intermediate area, *TuI* tuberal intermediate area, *RTuD* retrotuberal dorsal area, *TuD* tuberal dorsal area, *PPaV* peduncular paraventricular ventral nucleus, *DMH* dorsomedial hypothalamic nucleus, *VMHim* medial- intermediate VMH subnucleus, *vz* ventricular zone, *mz* mantle zone, *VMHil* lateral-intermediate VMH subnucleus, *OCh* optic chiasma

**Fig. 13:**
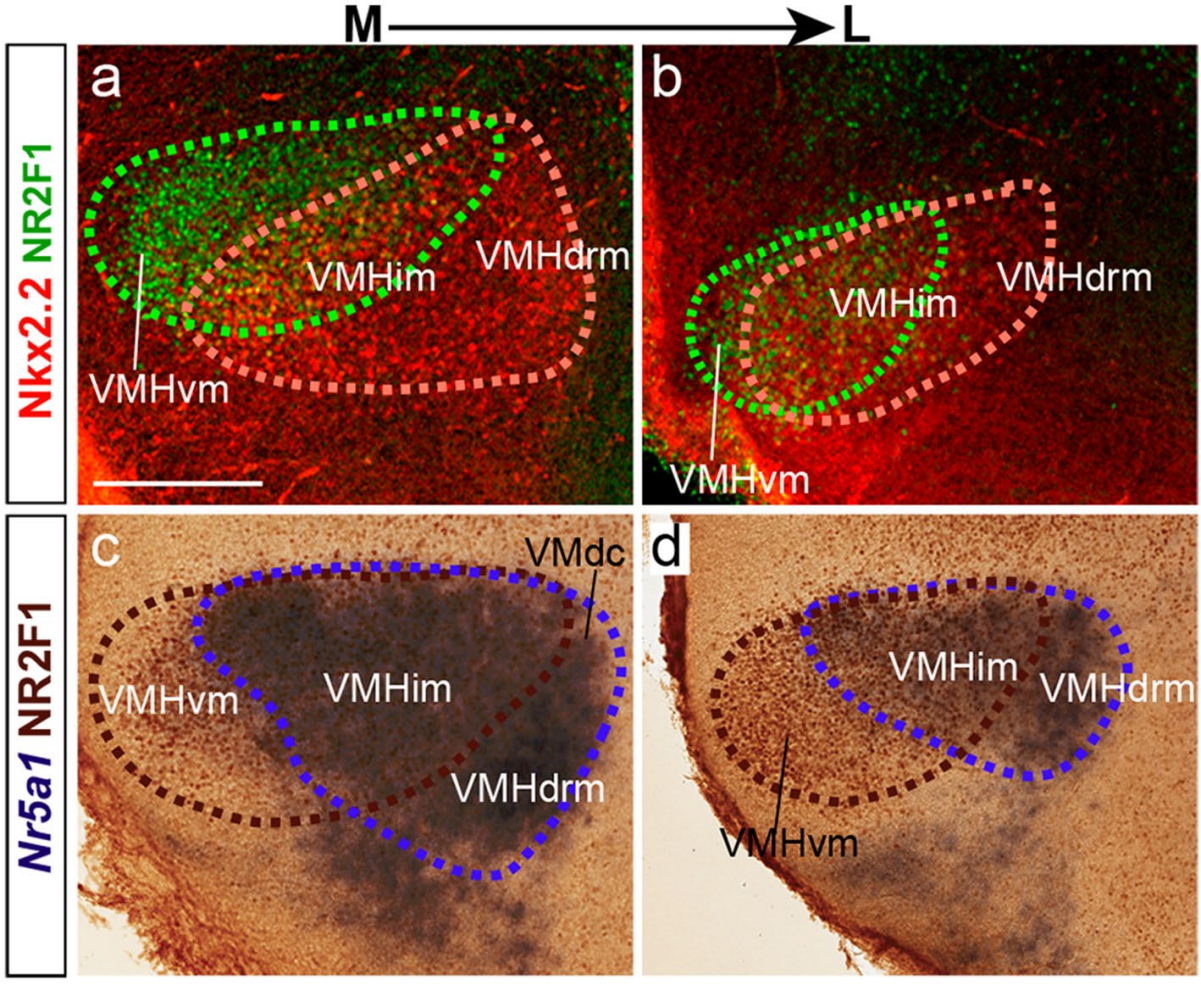
Nkx2.2, *Nr5a1* and Nr2f1 signals represent partially overlapping subpopulations of the VMH primordium. **a**, **b** Double immunofluorescence for Nkx2.2 (red signal; pink dash line) and Nr2f1 (green signal and dash line) in VMH sagittal sections at two section levels at E16.5, showing partial overlap. **c**, **d** Combined *Nr5a1* ISH (dark bluish signal; blue dash line) and Nr2f1 immunohistochemical reaction (brown signal and dash line) at two different section levels. There is also only a partial overlap of these two populations. Scale bars represent 200 µm. *VMHvm* ventral-medial VMH subnucleus, *VMHim* medial- intermediate VMH subnucleus, *VMHdrm* dorsal-rostromedial VMH subnucleus, *VMHdc* dorsocaudal VMH subnucleus

The *Nr2f1* marker (previously known as *Couptf1*) apparently labels a VMH subpopulation arising exclusively within TuI. This gene is expressed throughout the TuI/RTuI domains of the basal hypothalamus (excepting the corresponding acroterminal area) at E11.5 (not shown; see Allen Developing Mouse Brain Atlas) and E13.5 (Fig. 12a, b). The TuD/RTuD area where early expression of *Nkx2.2* and *Nr5a1* was observed is devoid of *Nr2f1* signal (Fig. 12a, b). At E15.5, *Nr2f1* signal appears both within the dorsomedial hypothalamic nucleus (DMH; hp1 + hp2) and the VMH (hp2; Fig. 12c, d). The latter formation shows two kinds of labeling: an abundant *Nr2f1* signal that marks particularly the ventral parts of the VMH primordium, and dispersed *Nr2f1* neurons within the prospective intermediate part of VMH (red and blue asterisks in Fig. 12b, d). Subsequently this marker appears strongly expressed at the VMHdc, and ventral portions of VMH. It also shows weaker expression at the transitional limits of the VMHim, VMHil, and VMHdrm subdivisions (Figs. 5i, 13a–d).

We compared with double immunofluorescence the distribution of Nkx2.2- versus Nr2f1-positive cells. At E12.5, the longitudinal Nkx2.2-positive hypothalamic progenitor territory appears in sagittal sections dorsal to the longitudinal domain marked by Nr2f1, with only a small area of overlap between them (Fig. 12e, e’). At E14.5 the VMH primordium contains both examined populations; Nkx2.2 cells are largely restricted to its dorsal part, whereas Nr2f1 cells appear mainly in its ventral part, with a small overlap visible in sagittal sections (Fig. 12f). Using horizontal sections oriented parallel to the prosomeric alar-basal boundary (see Fig. 1), the ventricular zone observed in the dorsalmost sections through basal hypothalamus (next to the alar-basal boundary) contains Nkx2.2-positive elements, but no Nr2f1 cells (Fig. 12g–g’). On the contrary, at levels through the TuI the ventricular zone shows Nr2f1 expression but no Nkx2.2 signal, though migrated Nkx2.2 cells are present in the mantle layer (Fig. 12h–h’). We also compared at E16.5 the Nr2f1 VMH subpopulation with Nkx2.2 or *Nr5a1*-marked cells. Although a degree of overlap exists between any of these populations, there are still indications of a partially differential dorsoventral distribution, with *Nr5a1* and Nkx2.2 cells occupying more importantly the VMHdrm subnucleus, and Nr2f1 cells preferentially appearing in ventral parts of VMH where the other two markers are absent (Fig. 13a–d).

#### Nkx2.1

**Fig. 14:**
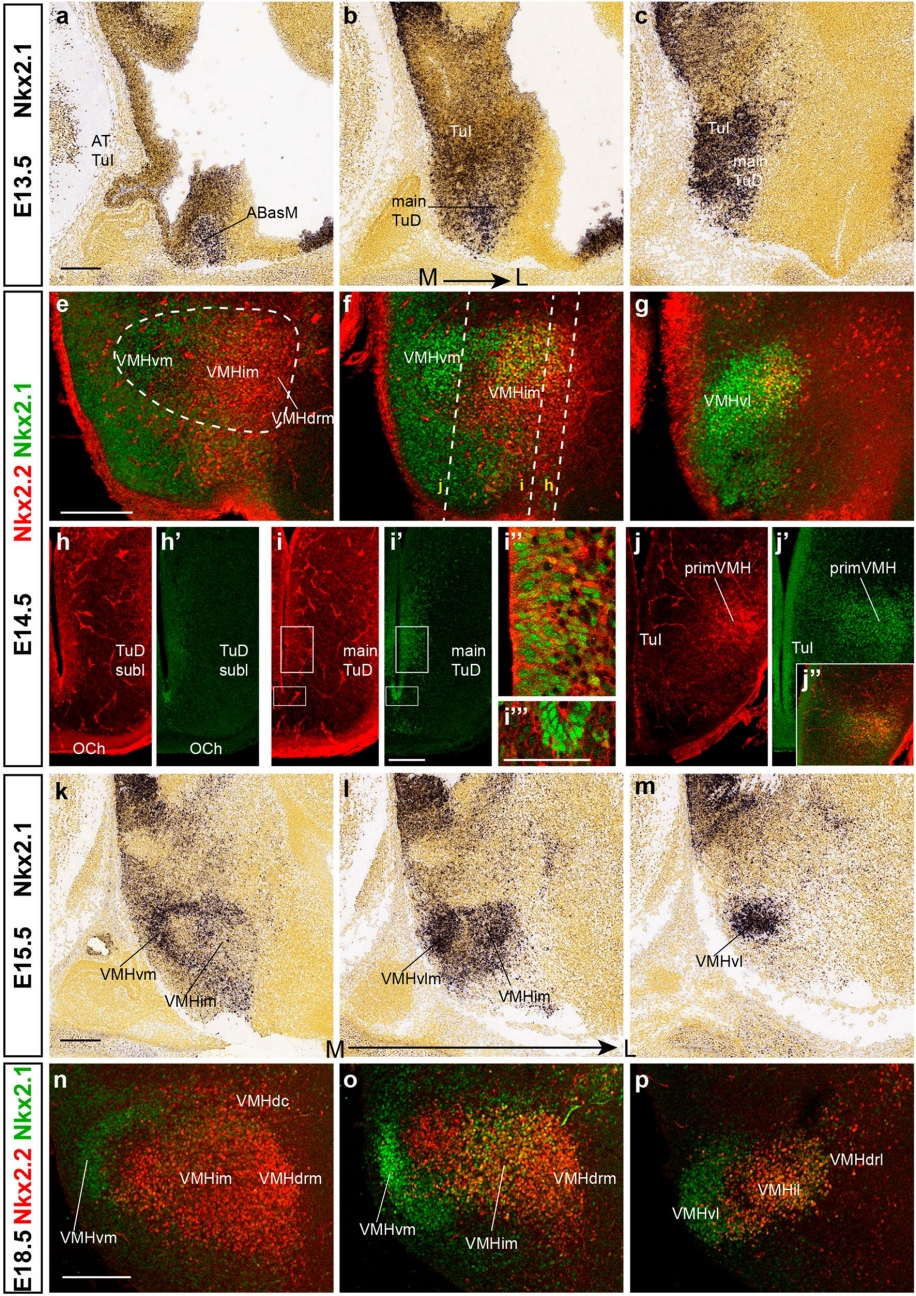
Widespread basal expression of *Nkx2.1* hypothalamus marker but selectivity at the VMH primordium. **a**–**c** Medio-lateral E13.5 sagittal sections from the Allen Developing Mouse Brain Atlas with *Nkx2.1* ISH show widespread basal signal of this hypothalamus marker (check also Fig. 3**h** in the adult). **e**–**g** Comparison of Nkx2.2 (red) and Nkx2.1 (green) immunofluorescence in mediolateral sagittal sections at E14.5. The dashed contour of the VMH primordium is indicated in (**e**). The labeled cell populations are mostly topographically distinct, though a few double-labeled cells are observed dorsocaudally (yellow in **f**, **g**). (**h**–**j**) Dorsoventral horizontal sections of E14.5 embryos reacted for Nkx2.2 (red) and Nkx2.1 (green) immunofluorescence (section levels marked in **f**). At level **h** there is a poor Nkx2.1 signal (**h**’); this starts to appear stronger at ventricular and periventricular zone **i** levels (**i’**, **i’’**; **i’’** and **i’’’**), and expands into the VMH mantle at **j** levels (**j’**, **j”**). **k**–**m** Medio-lateral sagittal sections at E15.5 from the Allen Developing Mouse Brain Atlas reacted for *Nkx2.1* ISH, showing preferent reaction at VMHim, VMHvm, and VMHvl subdivisions. **n**–**p** Comparison of double Nkx2.2 (red) and Nkx2.1 (green) immunofluorescence at mediolateral sagittal section levels from E18.5 embryos. Note double-labeled yellow cells at VMHim (**o**). Scale bars in **a**, **e**. **k**, **n** represent 200 µm. Scale bar in **i’** represents 100 µm. Scale bar in **i’’’** represents 50 µm. *AT* acroterminal area, *TuI* tuberal intermediate area, *TuD* tuberal dorsal area, *VMHvm* ventral-medial VMH subnucleus, *VMHim* medial-intermediate VMH subnucleus, *VMHdrm* dorsal-rostromedial VMH subnucleus, *VMHvl* ventral-lateral VMH subnucleus, *primVMH* primordium of the ventromedial hypothalamic nucleus, *VMHdc* dorsocaudal VMH subnucleus, *VMHil* lateral-intermediate VMH subnucleus, *VMHdrl*, dorsal-rostrolateral VMH subnucleus

*Nkx2.1* is widely expressed in the hypothalamic basal plate at early stages (starting at neural plate stages; Shimamura et al. 1995; Qiu et al. 1998) but is absent at the retromamillary area (RM) and its migrated VPM and STh derivatives (Puelles et al. 2012; López-González et al. 2021); there is also a thin longitudinal band of *Nkx2.1*-positive cells ventrally within the SPa alar area; this may correspond to its liminal subdomain and its origin remains uncertain (van den Akker et al. 2008; Puelles et al. 2012; LP, unpublished observations). At E13.5, *Nkx2.1* is strongly expressed in the ventricular and mantle zones of the TuD and TuI progenitor domains (Fig. 14a–c). Moreover, *Nkx2.1* marks also the whole basal acroterminal territory from E11.5 onwards (Allen Developing Mouse Brain Atlas data; Fig. 14a; Puelles et al. 2012, their Figs. 8.9D; 8.10D). At E15.5, the VMH primordium contains deep *Nkx2.1*-positive neurons in its ventral and dorsal parts (not so at the medial-intermediate part), and separate superficial cells are observed ventrally (Fig. 14k–m). At E18.5 the *Nkx2.1* signal largely occupies the VMHdc, VMHdrm (with slight extension into VMHdrl), VMHil VMHvm, and VMHvl subnuclei (Fig. 14n–p).

A comparison of Nkx2.1 and Nkx2.2 distribution at E14.5 corroborates the predominancy of Nkx2.1 at ventral loci of the VMH, whereas Nkx2.2 is placed mainly at intermediate levels, with some extension into the dorsocaudal subnucleus (Fig. 14e–g). In our horizontal sections (similar to conventional columnar coronal sections but interpreted along the axial dimension), we observe Nkx2.1 and Nkx2.2 cells in the ventricular stratum at TuD levels (Fig. 14h–i’’’). The presence of Nkx2.1-positive cells increased in the ventricular layer at TuI levels, whereas Nkx2.2 cells are totally absent in this stratum (Fig. 14j, j’). At these sections the VMH primordium in its entirety is revealed complementarily labeled by the Nkx2.2 and Nkx2.1 markers (Fig. 14j, j’, j’’).

#### Six3

**Fig. 15:**
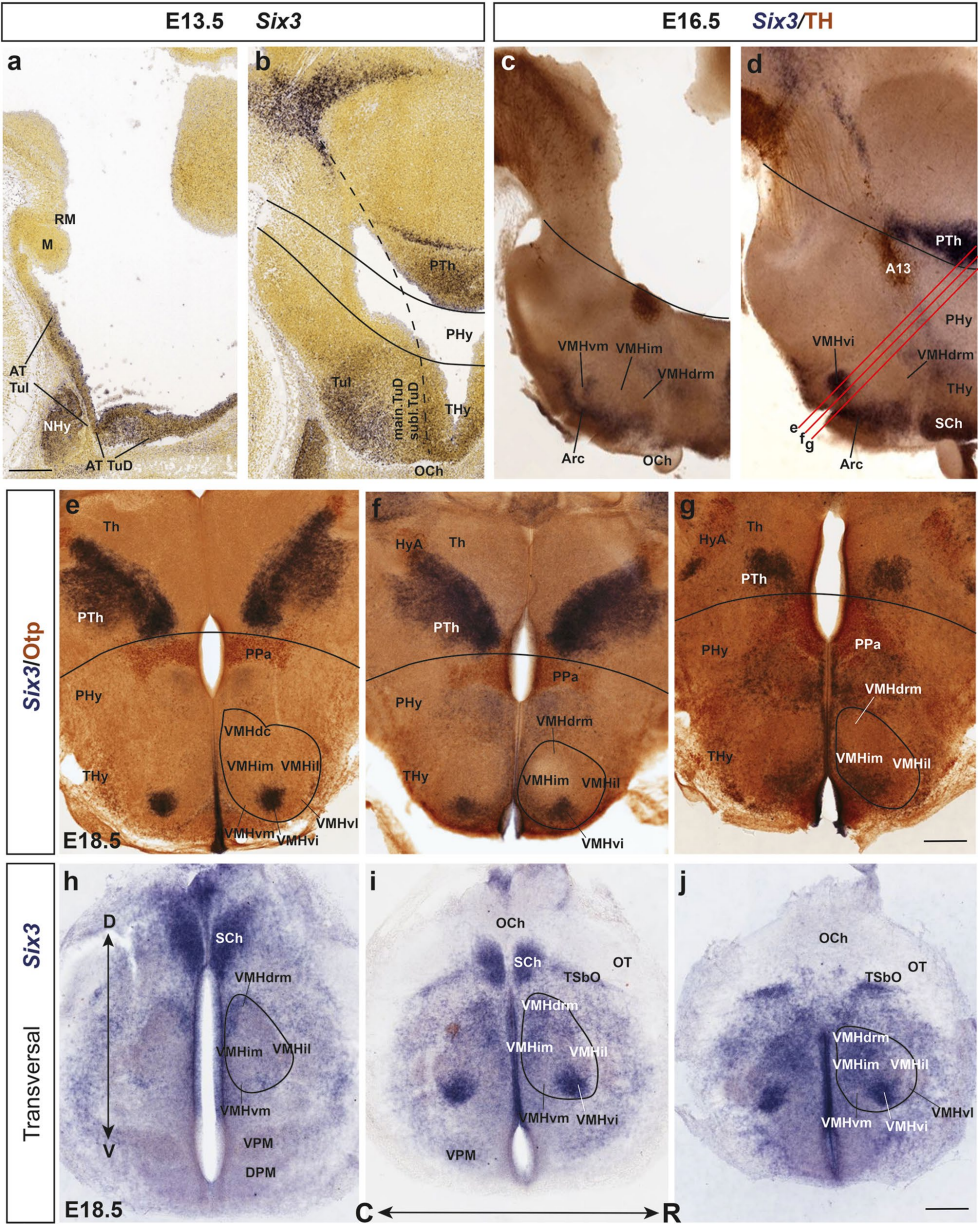
The early acroterminal marker *Six3* (see **a**) later reveals a distinct ventral VMH subnucleus (**d–f, i, j**). *Six3* ISH images from Allen Developing Mouse Brain Atlas at E13.5 (**a**, **b**) and E16.5 (**c**, **d**) illustrate that apart of *Six3* cells developing within the acroterminal Arc and SCh nuclei, a small compact ovoid mass appears labeled within the neighboring VMHvi subdivision as development advances. Parallel red lines in **d** represent the somewhat oblique section planes of sections **e**–**g**, which interest caudally the negative thalamus (Th) and *Six3*-positive prethalamus (PTh), apart of the alar Otp-immunopositive paraventricular nucleus (Pa) and hypothalamo-amygdalar corridor (HyA). **e**–**g** Oblique serial sections showing combined *Six3* ISH (blue) and Otp IHC (brown) expression in an E18.5 embryo, identifying the novel VMHvi subnucleus and additional more disperse similar cells in VMHim and VMHdrm. The black curved line represents the hypothalamo-diencephalic boundary, also shown in black in (**c**, **d**; note here the counterstain is TH, marking the A13 cell group). **h**–**j** *Six3* ISH caudorostral serial sections from an E18.5 embryo transversal to the basal hypothalamus (the optic chiasma –OCh– and suprachiasmatic nuclei –SCh– appear dorsally; TSbO, tuberal suboptic nucleus). The contour of the VMH complex is delineated and the intermediate ventral position of the novel Six3-positive VMHvi subdivision is evident. Scale bars represent 200 µm. *RM* retromamillary area, *M* mamillary area, *AT* acroterminal area, *NHy* neurohypophysis, *TuI* tuberal intermediate area, main or subl. *TuD* main or subliminal tuberal dorsal area, *PTh* prethalamus, *PHy* peduncular hypothalamus, *THy* terminal hypothalamus, *OCh* optic chiasma, *VMHvm* ventral-medial VMH subnucleus, *VMHim* medial-intermediate VMH subnucleus, *VMHdrm* dorsal-rostromedial VMH subnucleus, *Arc* arcuate nucleus, *VMHvi* ventral-intermediate VMH subnucleus, *A13* A13 dopaminergic cell population, *PTh* prethalamus, *SCh* suprachiasmatic nucleus, *Th* thalamus, *PPa* peduncular paraventricular area, *VMHdc* dorsocaudal VMH subnucleus, *VMHil* lateral-intermediate VMH subnucleus, *VMHvl* ventral-lateral VMH subnucleus, *VPM* ventral premamillary nucleus, *DPM* dorsal premamillary nucleus, *OT* optic tract, *TSbO* tuberal suboptic nucleus

This is a particularly distinct and previously undescribed partial marker of the VMH nucleus, where it identifies a new subdivision. Early on, *Six3* is strongly expressed at the acroterminal parts of TuD and TuI, the latter representing the primordium of the arcuate nucleus, median eminence and infundibulum (Fig. 15a). In contrast, the neighboring subliminal and main TuD domains are *Six3*-negative, as well as the incipient VMH primordium (Fig. 15b). At E16.5, though, strong *Six3* signal can be identified at the acroterminal arcuate and anterobasal nuclear primordia (basal plate) and the suprachiasmatic primordium (alar plate) while the VMH primordium shows a novel strongly *Six3*-positive ovoid subpopulation within its ventral-intermediate part (VMHvi; Fig. 15c, d). Subsequently, at E18.5, *Six3* continues labeling strongly the well delimited, ovoid mass seen previously (VMHvi); this is found intercalated between the molecularly different VMHvm and VMHvl subdivisions (VMHvi; 15e–j). A weaker and more dispersed expression of *Six3* is detected as well at the VMHim and VMHdrm subdivisions, whose labeled cells are less well delimited from the arcuate population, in contrast with the VMHvi (Fig. 15h–j).

## Discussion

The VMH nucleus is one of the larger hypothalamic structures (called ‘principal hypothalamic nucleus’ by Cajal). Classically it has been widely defined as presenting dorsomedial, central/core, and ventrolateral cytoarchitectonic subdivisions (VMHdm, VMHc, VMHvl). Note the corresponding topographic descriptor terms refer to the columnar forebrain axis postulated as running into the telencephalon (the prosomeric dorsal direction). The VMH massively contains glutamatergic neurons (Puelles et al. 2012; their Figs. 17A–C; 20A–C; 22, 23A,B). However, recent scRNAseq transcriptomic studies found an unexpected diversity of neuronal molecular profiles in the VMH (Kim et al. 2019 found 12 neuronal clusters in the core portion of the VMH and 17 neuronal clusters in the ventrolateral VMH part, whereas van Veen et al. 2020 described six VMH neuronal types). Affinati et al. (2021) identified 24 VMH clusters roughly categorized into 6 main groups. Comparison of diverse gene markers mapped in diverse section planes in the developing VMH already led Puelles et al. (2012) to the conclusion that VMH was heterogenous and might not be originated as a whole within the intermediate tuberal area (TuI), the subdivision of the prosomeric basal hypothalamus in whose terminal part (THy) the VMH lies in the adult. Evidence was presented suggesting the contribution to VMH at least of *Nkx2.2*- and *Pdyn*-expressing cells, which apparently originated from the overlying TuD progenitor domain (Puelles et al. 2012; their Figs. 26A–N). Unfortunately, this well-documented notion has been generally disregarded in the subsequent literature. Accrued reports on loss of function of selected VMH gene markers (Kurrasch et al. 2007; Cheung et al. 2013; Lu et al. 2013; Corman et al. 2018; Aslanpour et al. 2020a, b) did not contemplate the prosomeric AP and DV subdivisions of the tuberal hypothalamic region and the phenotypes observed were interpreted under the traditional (simpler) columnar assumptions (e.g., see Altman and Bayer 1978, 1986). These include the undocumented assumption that the whole VMH cell population is produced locally in the tuberal area (i.e., without any tangential migration; further comments on this below). Columnar reports on the VMH frequently concentrate descriptions on a sole coronal section level midways through the nucleus (which is implicitly interpreted as a transversal section and does not show the more peripheral parts of the tuberal area) and sagittal or true transversal sections of the VMH (see our Fig. 3a–g) are seldom examined. This leads to practical invisibility of our highly relevant TuD and acroterminal domains, the main sources of tangentially migrated VMH cells.

We have addressed this issue consistently with previous prosomeric analysis, correlating diverse molecularly characterized VMH neuronal populations to the set of molecularly defined progenitor domains previously reported in the area of interest (Puelles et al. 2012; review in Diaz and Puelles 2020; see also Morales-Delgado et al. 2011, 2014). We were able to map early emergence of some distinct VMH cell types in specific surrounding domains of the embryonic hypothalamus, and then traced them via intermediate stages to their ulterior topography within the VMH complex. These tracings suggested or were consistent with tangential or radial migration patterns according to the characteristic spatiotemporal transitions deployed in each case. Neurons that form the VMH proper (leaving aside the even more heterogeneous surrounding shell of the nucleus) were shown to originate mainly either in the TuD or TuI progenitor domains within basal THy, sometimes involving also or exclusively the corresponding TuD or TuI acroterminal subregions (see the novel prosomeric concept of the *acroterminal domain* as the rostromedian end of the THy in Puelles et al. 2012; Puelles and Rubenstein 2015; Ferran et al. 2015; Puelles 2018; Diaz and Puelles, 2020). A further contingent of VMH cells apparently originates from the overlying alar terminal subparaventricular area (TSPa), otherwise previously identified as a source of various dorsoventral peptidergic neuronal migrations from alar progenitor domains into the subjacent tuberal/retrotuberal basal plate (Diaz et al. 2015). Since the adult VMH apparently lies strictly within TuI, the TuD, acroterminal, and TSPa origins necessarily imply dorsoventral or rostrocaudal tangential migrations of the corresponding derivatives finally found inside the VMH. The existence of dorsoventral tangential cell translocations coming from the TSPa or TuD areas was verified experimentally.

**Fig. 16:**
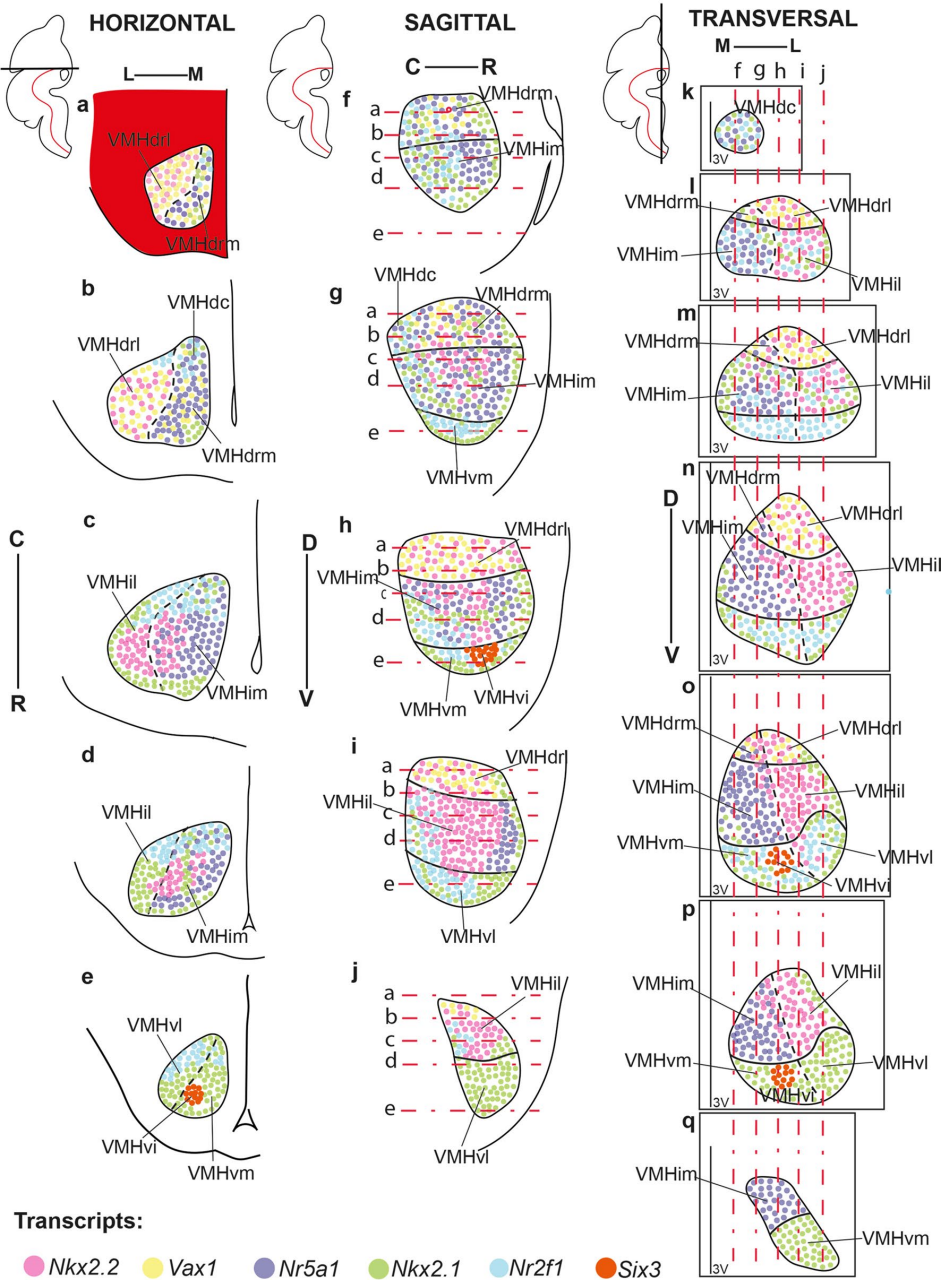
Schemata illustrating our updated VMH model with color-coded molecularly-typified populations. The schemata of successive VMH sections are arranged into horizontal **a**–**e**, sagittal **f**–**j** and transversal **k**–**q** series (see inset drawings above illustrating the section plane as a black line relative to the bent length axis of the prosomeric model, represented by the alar-basal boundary in red). The molecularly characterized cell groups mapped are represented as color-coded small circles. Note that these circles do not represent individual cells. Black dash lines in **a**–**e** and **k**–**q** separate the mediolateral halves of the VMH. Solid black lines in **f**–**q** delimitate the dorsal, intermediate and ventral parts of the VMH. Red dash lines in **f**–**j** show the rough position of horizontal sections **a**–**e**, and red dash lines in **k**–**q** show the positions of the sagittal section levels **f**–**j** in the transversal schemata. *VMHdrl* dorsal-rostrolateral VMH subnucleus, *VMHdrm* dorsal-rostromedial VMH subnucleus, *VMHdc* dorsocaudal VMH subnucleus, *VMHil*, lateral-intermediate VMH subnucleus, *VMHim* medial-intermediate VMH subnucleus, *VMHvl* ventral-lateral VMH subnucleus, *VMHvi* ventral-intermediate VMH subnucleus, *VMHvm* ventral-medial VMH subnucleus, *3 V* third ventricle

We also described the approximate final distribution of the specific markers we traced developmentally regarding different subdivisions or subnuclei identified in the E18.5 VMH. Due to the prosomeric approach, implying use of diverse axial references and positional landmarks (e.g., the longitudinal floor plate and the alar-basal boundary; or the transversal acroterminal domain and the intrahypothalamic boundary) and a related map of molecularly defined progenitor domains (Puelles et al. 2012; Puelles and Rubenstein 2015; Ferran et al. 2015; Diaz et al. 2015, 2020; López-González et al. 2021; Fig. 1a,b) in the context of 22 marker genes, our analysis of inner VMH structure was more detailed than was usual heretofore, inspiring and justifying the proposed terminological changes (Fig. 16; Table 3). VMH molecular regionalization along the non-arbitrary dorsoventral, rostrocaudal, and mediolateral (radial) dimensions was reexamined, leading to an expanded subdivision map of the VMH complex. This contains minimally 8 parts that are consistent with the existence of combinations of several neuronal populations that show differential molecular features, showing in their postmigratory configuration various degrees of partial overlap. The conventional simpler tripartite schema was approximately conserved by terminological distinction of *dorsal*, *intermediate*, and *ventral* VMH regions, subdivided as follows: our *dorsal* VMH region includes not only the conventional ‘dorsomedial’ part, whose topologic position is more precisely described as ‘dorsocaudal’ (VMHdc), but also a previously undescribed ‘dorsorostral’ part, which divides into *medial* and *lateral dorsorostral* subcomponents (VMHdrm, VMHdrl); our *intermediat*e VMH region roughly corresponds to the conventional ‘central or core’ part, but divides distinctly into *medial* and *lateral-intermediate* subunits (VMHim, VMHil); finally, our prosomeric *ventral* VMH region includes the conventional ‘ventrolateral’ part and displays distinct *medial*, *intermediate*, and *lateral* subunits (VMHvm; VMHvi; VMHvl). Our VMHdc indeed happens to be ‘relatively dorsal’ in the prosomeric VMH (i.e., close to the prosomeric alar-basal boundary of the tuberal area, like its VMHdrm/VMHdrl companions), and thus merits the ‘dorsal’ descriptor. The latter was previously widely used in reference to the seemingly obsolete *columnar axis* (check its inconsistence with the primarily *longitudinal Nkx2.2*-positive band, commented in Puelles and Rubenstein 2015, and note the wrong columnar assumption that coronal sections through the hypothalamus show the *dorsoventral* dimension, though they demonstrably show the *anteroposterior* one by passing caudalwards into the diencephalon and midbrain; Fig. 1c). The ‘medial’ descriptor in this term was changed to ‘caudal’ (VMHdm = VMHdc) because this term is not sufficiently selective; all dorsal, intermediate, and ventral parts of the VMH have ‘medial’ portions (i.e., parts closer to the periventricular stratum); this includes also the *dorsal-rostral* part of VMH that has a distinct medial half (VMHdrm). We reproduced the resulting structural schema in axially true transversal, sagittal, and horizontal section planes in Fig. 16, and we tried to clarify the new terminology for the readers in Table 3. The New Neuromorphology (Nieuwenhuys and Puelles 2016) made possible by modern molecular and transgenic experimental data claims the need to clarify various newly emerging concepts by carefully rationalized terminology changes. It is expected that full incorporation into usage of the new terms and concepts requires generational change, but progressive steps in that direction are absolutely necessary to the progress of neuroscience.

The first origin of *Nr5a1*- and *Nkx2.2*-positive neurons that eventually populate the VMHdc, VMHdrm, VMHdrl, VMHim. and VMHil subdivisions was traced to the early TuD area (differentially at its main ventral subdivision and its overlying subliminal *Nkx2.2*-positive part); these two TuD populations migrate respectively in deep versus superficial mantle cell streams, and later show a medial to lateral (radial) stratification (*Nkx2.2* cells born at the subliminal TuD migrate superficially and finally lie lateral/superficial to the population of *Nr5a1* cells born at the underlying main TuD and migrating in a deeper radial level). Both parallel cell populations massively migrate ventralwards (tangentially) as observed at E12.5-E14.5, thus forming transient separate deep and superficial streams that spread out within the upper dorsal and intermediate parts of the VMH primordium once they reach the TuI. Additional molecularly distinct VMH cell populations were traced instead to local origins within the TuI ventricular zone, which underlies radially the VMH primordium. These comprise at least cell types expressing *Nr2f1*, *Lmo4, Satb2* (Tables 1, 2). In some cases, a given molecular subtype may be produced at both the TuD and TuI domains (e.g., *Nkx2.1*, *Sox14*).

The novel dorsal-rostral VMH subdivisions (VMHdrm, VMHdrl; first described in the present report, though previously implied by some reported observations) apparently derive mainly from the acroterminal part of the TuD progenitor region (represented, e.g., by *Tcf7l2*, *Sox14*, and some rostral *Nr5a1 cells*); the migration into VMH of these components thus implies an oblique *dorso-rostrocaudal* directional vector.

The *Six3*-expressing cells of the VMHvi also arise within the acroterminal basal domain; it is open to discussion whether they come from the TuI or TuD acroterminal subareas, or from both. We suspect that the TuI acroterminal subarea is principally related to the production of arcuate nucleus neuronal types (in caudal continuity with the DM complex), where mainly GABAergic neurons are produced; this would suggest that the *Six3* cells of the VMH (also first described in the present report) may arise instead within the molecularly distinct acroterminal TuD (site of the prosomeric ‘anterobasal nucleus’, or classical ‘retrochiasmatic area’).

Finally, *Vax1*-expressing VMH neurons originate clearly within the overlying subparaventricular alar progenitor area (TSPa), where *Dlx* family genes are also strongly expressed and GABAergic cells are thus expected to arise. This observation of some potentially GABAergic neurons expressing *Vax1* and maybe *Dlx* genes within VMH (mainly at the VMHdrm and VMHdrl subnuclei) is unexpected and needs to be checked in appropriate material (see, for instance, Puelles et al. 2012; their Figs. 8–18 and 8–15C, D, which seem to support this conclusion).

### Both TuI and TuD are progenitor areas for VMH cells

Topographically, the development of VMH seems linked to the *basal region* of the terminal hypothalamus (THy), namely to its tuberal territory (Tu). Nothing similar to the VMH develops within the cognate RTu area of the peduncular hypothalamus (PHy) (in prosomeric theory, THy and PHy belong to different prosomeres -hp2 and hp1, respectively- and Tu/RTu have therein analogous topologically basal positions under the alar-basal boundary; they are thus presumed to result both from similar ventralizing versus dorsalizing antagonism occurring in  two molecularly distinct neuromeres, thus the observed differences; the existence of important typological neuronal differences between these two hypothalamic areas is both unsuspected and inexplicable within the obsolete now more than 100 years-old columnar frame of thought). The unique developing VMH structure is accordingly one of the many differential features that can be explained by the postulate of the dual hp1 and hp2 hypothalamo-telencephalic prosomeres (Puelles et al. 2012; Ferran et al. 2015) but has no explanation within the alternative columnar system. The ample tuberal domain of THy can be divided dorsoventrally (according to the prosomeric axis) on the basis of molecular and structural features into dorsal (TuD), intermediate (TuI) and ventral (TuV) progenitor domains. All three of them extend rostralwards into the corresponding parts of the rostromedian acroterminal area (AT; Puelles et al. 2012). TuI is the topographic site of both the terminal part of DMH and the VMH, with the terminologically unacceptable oddity that the terminal DMH component clearly lies *ventral* to VMH (this topologic incongruency of the columnar names was already underlined by Puelles et al. 2012 but was not noticed when these columnar names were introduced, probably because it was assumed that the terminal DMH component was as a whole *caudal* to the VMH, which is maybe slightly less incongruent in columnar terms, but is in any case equally false in prosomeric terms; see also Puelles 2019). In contrast to the VMH that is restricted to TuI, the DMH complex of RTuI is bineuromeric (having terminal/hp2 and peduncular/hp1 moieties). This DMH longitudinal continuity passing finally *under* the VMH (in the prosomeric schema) already suggests that something extraordinary *linked only to THy and hp2* affects the development of VMH. Given that the DMH formation is largely GABAergic and essentially locally produced (with minor immigrated glutamatergic core nuclei; Puelles et al. 2012), it might be easily deduced that perhaps the massively glutamatergic VMH subarea of TuI is not really a part of TuI, but a variant subdomain of terminal Tu intercalated between TuD and TuI which may have unique properties. Based on the identification within TuD of the earliest expression of the *Nkx2.2* and *Nr5a1* markers which later characterize some positionally distinct types of VMH cells, Puelles et al. (2012) estimated that at least some VMH neurons possibly originated via selective dorsoventral tangential migration from the terminal TuD domain. At that time, it was unclear whether any VMH neurons arise primarily at TuI level. Puelles et al. (2012) rather inclined to the assumption that *all* VMH subpopulations might be migrated from TuD.

We reexamined this issue with more abundant molecular data and some experimental testing of the previously predicted dorsoventral migration, corroborating its existence, but reaching the conclusion that the mouse VMH apparently contains both local and exogenous neuronal populations. Some neurons with differential molecular profiles can be traced to origins within either TuI or TuD, separately, or jointly. Moreover, additional VMH subpopulations were found that seem to originate specifically in the basal AT area lying dorsal to the Arc area, and some neurons migrate into VMH coming from the overlying alar subparaventricular area. This supports the idea that patterning of the tuberal area occurs differentially at PHy compared to THy, introducing the unique and complex VMH areal phenomenon found dorsal to the terminal end of the DM ‘column’.

The contextual background of any neural developmental analysis includes data on local neurogenesis. Reportedly, VMH neurogenesis occurs in mice between E9.5 and E14.5, with a peak at E11.5-E12.5 (Shimada and Nakamura 1973; Aslanpour et al. 2020b; similar rat data in Ifft 1972 or Altman and Bayer 1986). Interpreting their data in terms of the columnar model, Aslanpour et al. (2020b) identified a “rostrocaudal” gradient in VMH birthdates, which we translate topologically to the prosomeric model as a *dorsoventral gradient*. Presumably TuD neurogenesis precedes that of TuI, which is consistent with reported wholemount rat results using AChE as an early differentiation marker (Puelles et al. 2015; Amat et al. 2022). Accordingly, we deduce that our dorsal tuberal *Nr5a1* and *Nkx2.2* VMH cells would be born earlier (E10.5) than the ventral ones (E11.5), due to their origin in the more precocious TuD area.

### Dorsally originated Nkx2.2 cells invade the intermediate VMH via tangential migration

Descriptive analysis of gene patterns led Puelles et al. (2012) to suggest the migration *en masse* into VMH of *Nkx2.2* elements born at the subliminal dorsal tuberal area that underlies the alar/basal limit. We confirmed this migratory process with our own descriptive and experimental data. We collaterally noted that less numerous ventrally migrating *Nkx2.2* cells from the subliminal dorsal retrotuberal area seem to invade the area of the peduncular dorsomedial hypothalamic nucleus, though they are not clearly detectable at advanced embryonic stages (Puelles et al. 2012; Kim et al. 2021). We also observed migration of CMFDA-labeled Nkx2.2 cells into the VMH primordium when the TuD area under the alar-basal border was marked.

The TuD area is divided into dorsal subliminal and ventral main portions; the migrating *Nkx2.2* cells are thought to arise at the subliminal part, so that they must cross the underlying main part to reach the VMH. The main TuD domain is where *Nkx2.1-*positive *Nr5a1*-expressing cells are produced. The subliminal TuD portion reportedly does not express either *Nkx2.1* or *Nr5a1*. The molecular pair *Slit*/*Robo* involved in regulation of neuronal migration and axonal guidance is expressed at the VMH primordia. The transcription factors *Nkx2.1*/*Isl1*/*Otp* regulate *Robo2* expression. Moreover Slit1–/– and Slit2–/– mice show increased cell density at the VMH subventricular zone (Romanov et al. 2020). It would be interesting to analyze whether the *Slit*/*Robo* signaling pathway affects the migrating *Nkx2.2* population of VMH.

The transcription factor *Nr5a1* (SF1) plays a role in VMH development regarding its neuronal identity (Tran et al. 2003), cell distribution (Ikeda et al. 1995; Shinoda et al.^1995^; Davis et al. 2004) and connectivity (Tran et al. 2003). This factor was widely used in functional studies of the VMH nucleus (Büdefeld et al. 2012; Hashikawa et al. 2017; Kennedy et al. 2020; Lewis et al. 2022). In contrast, there is little information about the role of the *Nkx2.2* transcription factor in VMH development (Kurrasch et al. 2007; Puelles et al. 2012). In this study we have compared the developmental distribution of both, *Nr5a1* and *Nkx2.2*, noting that, irrespective of partial overlaps, they largely occupy different sectors of the VMH. The literature often wrongly assumes that *Nr5a1* is a *general* *transient* marker of the VMH, but that is certainly not the case, according to our data. *Nr5a1* cells occupy a larger part of VMH than *Nkx2.2* ones, but both are restricted to given VMH subnuclei (*Nr5a1* mainly at VMHdc, VMHdrm and VMHim; *Nkx2.2* is found in a mediolateral gradient along the intermediate VMH tier, being stronger at its lateral part, in contrast with the medially predominant *Nr5a1* subpopulation). This pattern is thus explained by our interpretation that both populations are exogenous relative to the VMH area and each invades it differentially according to different origins, routes, and final distributions (presumably affected by their differential reaction to the local adhesivity or attractor scenario found within the VMH).

The *Nr5a1* pattern across AT/main TuD/TuI-VMHdc/VMHdrm/VMHim is reproduced by other markers (e.g., *Cnr1*, *Slc17a6*, *Robo2*), whereas the characteristic *Nkx2.2* pattern across subliminal-TuD-VMHil/VMHdrl is rather unique.

### TuI origin of some VMH neurons

Radial migration has been assumed conventionally as the only mechanism laying down the VMH primordium in the tuberal mantle. Such cells would move from the local ventricular zone into the neighboring mantle, presumably guided by outside-in stratification rather that radial migration proper (McClellan et al. 2006). Misplaced cells containing estrogen receptors identified in *Nr5a1* null mice or in *GABA*_*B*_*R1* knockout mice have been interpreted to represent failed radial migration (Dellovade et al. 2000; McClellan et al. 2008). Moreover, agonists and antagonists of GABA_A_ and GABA_B_ receptors acting in vitro on coronal hypothalamus slices (these are actually horizontal relative to the hypothalamic basal plate) were reported to change the speed and orientation of radially migrating neurons (Dellovade et al. 2001; McClellan et al. 2008). In all these cases the authors did not consider the possibility that processes involving tangential migration might be affected instead or simultaneously.

The conventional ‘ventrolateral’ VMH subregion, which apparently is majoritarily colonized by radially migrated neurons, is well known for the selective mapping of estrogen receptors (e.g., Cheung et al. 2013; Krause and Ingraham, 2017; Ma et al. 2021). Some authors described also *Nkx2.1* expression as highly restricted to ‘ventrolateral’ VMH (Tran et al. 2003; Davis et al. 2004; Cheung et al. 2013). Our data indicate that early *Nkx2.1* expression distributes widely throughout the tuberal region. At E18.5 we still observe expression of this marker at the tuberal periventricular stratum deep to the VMH, as well as in neurons of some VMH subdivisions, namely the VMHdc, VMHvm, and VMHvl subnuclei. Other radially migrated local populations studied by us are mainly found at the ventral portion of the VMH. This is the case of *Nr2f1* cells identified eventually at the ventral VMH tier (VMHvm, VMHvl), though some of them overlap with tangentially migrated *Nr5a1* and *Nkx2.2* elements in VMHim/il, respectively. *Nr2f1* cells also appear separately at the VMHdc subnucleus (=conventional VMHdm subdivision). Similarly, the *Satb2* marker identifies another radially migrated local cell population found within the ventral VMH tier (VMHvm, VMHvl), which is differentially absent from the intermediate tier. These observations suggest that also the radially migrated components of VMH may have subtly diverse topographic origins and postmigration distributions.

### Varied neuronal identities within the VMH

Lu et al. (2013) showed evidence suggesting that the early transcription factor *Rax* has a role in conferring a *Nr5a1*-expressing and glutamatergic cell fate to cells in the central VMH. *Rax* is an early gene expressed at the TuI and TuD (our interpretation of Allen Developing Mouse Brain Atlas material at E11.5 and E13.5), overlapping the *Shh*-positive basal plate territory, while a large part of the rostral acroterminal domain (particularly the TuI one) loses secondarily the *Shh* signal. We noted that the prospective VMH area within TuI retains up to E13.5 a central patch of low level *Shh* signal. Lu et al. (2013) observed that when *Rax* was floxed under control of the *Shh* promotor, a central region of *Nr5a1 *cells within VMH lost this signal (among others related to the glutamatergic phenotype- see their Figs. 7M–P; 7A’, F’, B’) and came to be occupied by GABAergic neurons (their Fig. 7G’). Remarkably, surrounding dorsocaudal and ventral *Nr5a1* cells of the VMH remained unaffected. The authors illustrated the tuberal expression of *Shh* at E10.5 in a true transversal section (note the eye stalks and telencephalic vesicles lying above the hypothalamus) but did not identify this area as the TuD subregion (a longitudinal band extending under the alar-basal boundary); this is the dorsal basal domain that selectively retains strong primary *Shh* signal normally (Andreu-Cervera et al. 2019; Puelles et al. 2012). If this curious phenotype change occurring in the central or intermediate part of VMH depends on floxing that selectively occurred at the longitudinal *Shh*-positive TuD domain, the whole terminal TuD area producing tangentially migrating *Nkx2.2* and *Nr5a1* VMH neurons would be expected to have been affected. It is thus difficult to see why some parts of VMH developed normally. It is actually somewhat unclear in this report whether Rax is expressed strongly enough at the level of the TuD band, to respond to the local *Shh* floxing effect (see in this regard the results of Orquera et al. 2016, suggesting that there is little *Rax* expression within TuD).

A more detailed study of the Allen coronal series for E11.5 and E13.5 indicated that ventral to the strongly *Shh*-positive TuD band and wholly inside the *Rax*-positive domain there also appears *a patch of weaker Shh expression* which can be ascribed to the upper TuI area (VMH primordium), whose radial derivatives might contribute selectively to the central VMH portion that results devoid of *Nr5a1* cells and abnormally populated by GABAergic neurons (Lu et al. 2013). We therefore consider it likely that the observed restriction to central VMH of the Rax lack of function phenotype may be explained as due to an effect on the local patch of weak TuI *Shh* signal. Lu et al. (2013) did not show images of the TuD area above the VMH at later stages, which might have indicated whether the *Nr5a1* cells that normally migrate dorsoventrally into VMH may have failed to do so, displaying instead abnormal positions or signals of cell death. Moreover, when *Rax* was deleted under the action of the *Six3* promoter (a gene primarily overlapping *Nkx2.1* expression only at the acroterminal Arc area), *Nkx2.1* was also dramatically diminished in the VMH primordium (Lu et al., 2013). These results imply that some VMH cells are produced radially at the TuI area (under *Shh* control) and others derive tangentially from the acroterminal TuI area (under *Six3* control; it should be investigated whether this bears particularly on our novel *Six3*-positive VMHvi subnucleus). Both processes may require independently additional *Rax* function. It would be interesting to examine similarly whether *Rax* affects the *Nkx2.2-*positive population of the VMH.

Aslanpour et al (2020a) suggested that *Ascl1*, a bHLH transcription factor expressed early on in the ventricular stratum of the TuD and TuI domains, likewise switches on differentiation programs that give rise preferentially to some VMH neurons (e.g., our intermediate or dorsocaudal VMH populations).

Our results indicate that VMH neurons originate from diverse progenitor areas, and occupy characteristic, partly overlapping parts of the VMH. Hypothetic areas of overlap between the diverse progenitor domains might explain the occasional visualization of double-labeled VMH cells that express different combinations of transcription factors (e.g., *Nkx2.2*-*Nr5a1*, *Nkx2.2*-*Nkx2.1*, *Nkx2.2*-*Nr2f1*), besides cells that express *Nkx2.2* without *Nr5a1*, *Nkx2.1* or *Nr2f1*. Similarly, Corman et al. (2020) identified *Shh* cells and Shh-responsive (*Gli1*) lineage cells as contributing to the VMH population; they studied several VMH markers and quantified that, approximately, a half of *Nr5a1* cells, a half of *Nkx2.1* cells and 20% of *Nkx2.2* cells belong to a *Shh* lineage occupying a rostral VMH subregion, whereas *Gli1* lineage cells represent a proportion of caudal VMH cells, with relatively less *Nr5a1* and *Nkx2.1* elements, and more *Nkx2.2* neurons. These results agree with ours as regards the existence of molecularly diverse domains of origin that produce qualitatively different VMH subpopulations.

Studies examining loss of function of single transcription factors, or a morphogen such as *Shh*, have in common that, while a discrete part of the VMH population is affected, in none of them the whole nucleus is disrupted. Consistently with our present results, this suggests that there are several neighboring but different sites of origin of VMH cells, apparently with subtle variations in their transcriptomic set. Different subpopulations arising outside the radial position of the VMH (upper TuI) converge via parallel tangential migrations into the nuclear primordium. Once there, they partly disperse and intermix with other VMH cell types, without achieving a fully homogeneous distribution. This eventually results in the characteristic cellular typological profile of parts of the nucleus (Fig. 16; VMHdc, VMHdrm, VMHdrl, VMHim, VMHil, VMHvm, VMHvi and VMHvl), with different neurons, transcriptomic profiles, connections, and functions. We think that birthdating results, as well as most analyses of lack of function phenotypes, are so far non illuminating, because they were planned and interpreted assuming a single origin of the heterogeneous VMH neurons.

Indeed, recent single-cell transcriptomic studies have increased the ability to identify different VMH cell types. The available data on the hypothalamus already has made us realize that the number of distinct cell types was being conventionally vastly underestimated in the hypothalamus. In one of these studies, six principal VMH clusters were identified in postnatal mice (P10), each of them represented by a particular transcription factor: *Tac1*, *Rprm*, *Pdyn*, *Sst*, *Hpcal1*, *Galanin* (Veen et al. 2020). *Tac1* appears in the VMHdrm and VMHdrl subdivisions. In contrast, *Pdyn* is present at the intermediate VMH subdivisions, which makes plausible a phenotypic correspondence with the *Nkx2.2* cell phenotype, though further studies would be needed.

A more resolutive single-cell transcriptomic analysis identified 29 neuronal types within VMH, 17 of them apparently restricted to the conventional ‘ventrolateral’ VMH (Kim et al. 2019). These results show that the conventional columnar schema does not explain the existence of 29 different glutamatergic cell types within its rather simplistic (tripartite) VMH subdivision concept. After reinterpretation of such data within our present prosomeric VMH model we first note that the conventional ‘ventrolateral VMH’ only grossly corresponds to our *ventral VMH region*, whose demonstrated subdivision into at least VMHvm, VMHvi, and VMHvl parts was not known to columnar-based authors. Accordingly, it would be interesting to re-map the given set of 17 ‘ventrolateral’ clusters relative to our three ventral VMH subdivisions. Remarkably, Kim et al. (2019) identified a *Six3*-expressing cell cluster and they mapped it specifically to the ‘ventrolateral’ VMH region (their *Satb2_Six3* cluster disclosed by SMART-Seq); this entity most probably corresponds to our novel *Six3*-positive VMHvi subdivision. In this way, Hashikawa et al. (2017) identified a functional division of the ‘ventrolateral’ part regarding with differential mating and fighting behaviors. These data support the split of the classical ‘ventrolateral’ subdivision into the three subnuclei; VMHvm, VMHvi, and VMHvl that we propose in our present VMH model.

Affinati et al. (2021) similarly identified 24 molecularly differentiable cell clusters in the VMH, which they were able to categorize into 6 groups using majoritarily shared gene markers. According to these authors, *Dlk1* and *Esr1* expressing neurons are present at ‘ventrolateral’ classic division, *Lepr* expressing cells are located into the ‘core’, and Foxp1-cells map at their “anterior and lateral” to VMH core population, possibly corresponding to our VMHdrl subnucleus.

It seems probable, nevertheless, that even our more detailed VMH subdivision model is surpassed by these single-cell transcriptomic data, in the sense that all detected clusters cannot be ascribed uniquely to our specific VMH subdivisions. This means that our present minimally subdivided model probably also is inadequate in the long run to fully explain the real VMH populational complexity. Our present attempt to introduce the idea of alternative progenitor sources (and converging tangential migrations) for several components of the whole VMH population thus cannot be definitive and just points the way for future more detailed studies (e.g., each of our postulated progenitor domains may be subdivided). Interestingly, we also reached similar conclusions in two other previous studies, namely in the analysis of multiple progenitor domains (and mixed tangential migrations) contributing different neuron types to the prepontine interpeduncular complex (Lorente-Cánovas et al. 2012), as well as in our study of the tangentially migrated basal hypothalamic ventral premamillary nucleus (López-González et al. 2021).

## Conclusion

In this study we have addressed the identification and description of some molecularly identified VMH cell populations during development using immunofluorescence, in situ hybridization, and data extracted from the Allen Developing Mouse Brain Atlas. In our detailed descriptive analysis of these populations, we found important differences as regards their rostro-caudal and dorsoventral distribution throughout the VMH nucleus, which also partly change over time, suggesting in some cases the existence of tangential migration phenomena. These differences have not been described before due to the widespread use of a (single) coronal section plane in the analysis of this nucleus, which does not help the comprehensive interpretation of this territory, particularly when misinterpreted as cross-sections under columnar tradition. Many authors working on the VMH seem to be unaware that this nucleus is a basal plate derivative of only one of the two hypothalamic neuromeres (terminal hypothalamus; Puelles et al. 2012), and its possible patterning relationships with the hypothalamic floor plate and the alar-basal boundary (García-Calero et al. 2008; Andreu-Cervera et al. 2019), or the acroterminal (prechordal) area of influence, remain likewise unfocused, because these notions are disregarded by the obsolete columnar model. Using the interpretive conceptual apparatus offered by the prosomeric model, we showed that there exists a clear correspondence between several distinct progenitor areas (e.g., alar terminal SPa, and basal main TuD, subliminal TuD, TuI, TuD-AT, TuI-AT) where different VMH cell types are born and ulteriorly transferred via convergent tangential or radial migration to the developing VMH nucleus. This provides a new example of the utility of the prosomeric model to explain the organization of complex brain structures. Given that the new single-cell transcriptomic tools are manifestly sensible to the morphologic models used to map the characteristic clusters they detect, novel alternative interpretive systems need to be taken into consideration and tested as regards their possible advantages. A comprehensive and understandable molecularly defined cell type census over development and consequent further study of the respective mature cell populations can be significantly improved by searching optimal structural and developmental models.

We focused a good part of our analysis on the *Nkx2.2* transcription factor because it is related in origin to the alar/basal hypothalamic boundary, a not very well understood limit (some expert voices define it arbitrarily as an ‘oblique’ landmark; e.g., Shimogori et al. 2010), and also because it is a forgotten gene marker in most studies of VMH. We confirmed in organotypic cultures the related dorsoventral tangential migration that transfers a distinct *Nkx2.2* cell population from the dorsal tuberal region to the intermediate tuberal region, forming part of the intermediate part of VMH as suggested by Puelles et al. (2012).

## Data Availability

The original contributions presented in the study are included in the article, further inquiries can be directed to the corresponding author. Images from the Allen Developing Mouse Brain Atlas (https://developingmouse.brain-map.org/) were employed in this work.

## Abbreviations

ABasM: Medial (acroterminal) part of the anterobasal nucleus
ABasW: Wing (terminal) part of the anterobasal nucleus
A/B: Alar/basal limit
AP: Anteroposterior dimension
Arc: Arcuate nucleus
AT: Acroterminal region
Di: Diencephalon
DMH-P: Peduncular part of the dorsomedial hypothalamic nucleus
DMH-T: Terminal part of the dorsomedial hypothalamic nucleus
DV: Dorsoventral dimension
FP: Floor plate
hp1: Hypothalamo-telencephalic prosomere 1
hp2: Hypothalamo-telencephalic prosomere 2
HyA: Hypothalamo-amygdalar corridor
IF: Immunofluorescence
IHC: Immunohistochemistry
ISH: In situ hybridization
M: Mamillary area
ME: Medial eminence
NHy: Neurohypophysis
OCh: Optic chiasma
p2: Prosomere 2
p3: Prosomere 3
PHy: Peduncular hypothalamus
PM: Perimamillary area
PoA: Preoptic area
PPa: Peduncular paraventricular area
PPaV: Peduncular ventral paraventricular nucleus
PRM: Periretromamillary area
PSPa: Peduncular subparaventricular area
PTh: Prethalamus
RM: Retromamillary area
RTuD: Dorsal retrotuberal area
RTuI: Intermediate retrotuberal area
RTuV: Ventral retrotuberal area
SCh: Suprachiasmatic nucleus
Subl: Subliminal band
Tel: Telencephalon
Th: Thalamus
THy: Terminal hypothalamus
TPa: Terminal paraventricular area
TSPa: Terminal subparaventricular area
TuD: Dorsal tuberal area
TuI: Intermediate tuberal area
TuV: Ventral tuberal area
TSbO: Tuberal suboptic nucleus
VMH: Ventromedial hypothalamic nucleus
VMHdc: Ventromedial hypothalamic nucleus, dorsocaudal part
VMHdrl: Ventromedial hypothalamic nucleus rostrolateral dorsal part
VMHdrm: Ventromedial hypothalamic nucleus rostromedial dorsal part
VMHil: Ventromedial hypothalamic nucleus, intermedio-lateral part
VMHim: Ventromedial hypothalamic nucleus, intermedio-medial part
VMHvi: Ventromedial hypothalamic nucleus, ventro-intermediate part
VMHvl: Ventromedial hypothalamic nucleus, ventrolateral part
VMHvm: Ventromedial hypothalamic nucleus, ventromedial part
